# Prophage-like elements present in *Mycobacterium* genomes

**DOI:** 10.1186/1471-2164-15-243

**Published:** 2014-03-27

**Authors:** Xiangyu Fan, Longxiang Xie, Wu Li, Jianping Xie

**Affiliations:** Institute of Modern Biopharmaceuticals, State Key Laboratory breeding base of Three Gorges Eco-environment and Bioresources, Eco-Environment Key Laboratory of the Three Gorges Reservoir Region, Ministry of Education, School of Life Sciences, Southwest University, 400715 Chongqing, China

**Keywords:** Prophage, Mycobacterioprophage, Phylogeny, Comparative genomics

## Abstract

**Background:**

Prophages, integral components of many bacterial genomes, play significant roles in cognate host bacteria, such as virulence, toxin biosynthesis and secretion, fitness cost, genomic variations, and evolution. Many prophages and prophage-like elements present in sequenced bacterial genomes, such as *Bifidobacteria*, *Lactococcus* and *Streptococcus*, have been described. However, information for the prophage of *Mycobacterium* remains poorly defined.

**Results:**

In this study, based on the search of the complete genome database from GenBank, the Whole Genome Shotgun (WGS) databases, and some published literatures, thirty-three prophages were described in detail. Eleven of them were full-length prophages, and others were prophage-like elements. Eleven prophages were firstly revealed. They were phiMAV_1, phiMAV_2, phiMmcs_1, phiMmcs_2, phiMkms_1, phiMkms_2, phiBN42_1, phiBN44_1, phiMCAN_1, phiMycsm_1, and phiW7S_1. Their genomes and gene contents were firstly analyzed. Furthermore, comparative genomics analyses among mycobacterioprophages showed that full-length prophage phi172_2 belonged to mycobacteriophage Cluster A and the phiMmcs_1, phiMkms_1, phiBN44_1, and phiMCAN_1 shared high homology and could be classified into one group.

**Conclusions:**

To our knowledge, this is the first systematic characterization of mycobacterioprophages, their genomic organization and phylogeny. This information will afford more understanding of the biology of *Mycobacterium*.

**Electronic supplementary material:**

The online version of this article (doi:10.1186/1471-2164-15-243) contains supplementary material, which is available to authorized users.

## Background

Phages can be divided into virulent or temperate based on their relationship with the host. Temperate phage inserts and integrates into its host genome upon infection, and can reside as quiescent prophage. Prophage does not infect its host and maintains the dormant state [1]. Whole-genome sequencing reveals that prophage DNAs are widespread among bacterial genomes, even up to 20% of the host genome content [2]. Prophages are important genetic components transferred horizontally that can impart bacterial genome variability, evolution, and virulence [1, 3]. Some prophage genes contribute to the adaptation of bacteria to their specific ecological niches [3]. This has been demonstrated in many bacteria [1, 4, 5], but a little is known for *Mycobacterium* prophages.

There is huge gap between the number of mycobacteriophages isolated and cognate prophages found within mycobacteria. To date, there are 3427 mycobacteriophages isolated and 448 of them with genome sequenced. They can be assembled into 20 clusters (A-T) and seven of them are singletons [6, 7]. In contrast with large number of sequenced mycobacteriophages, their cognate prophages are poorly defined. Only the following mycobacterioprophage sequences have been described. Two prophage-like elements, phiRv1 and phiRv2, have been detected in *Mycobacterium tuberculosis* H37Rv genome [8]; two prophage-like elements, PhiMU01 and PhiMU02, are found within *M. ulcerans* Agy99 genome [9]; 10 putative prophages, named phiMmar01–10, are found in *M. marinum* M and two of them, phiMmar02 and phiMmar08, are full-length prophages [10]; the *M. abscessus* ATCC 19977 chromosome contains a full-length prophage and three prophage-like elements [11]; prophage Araucaria is found in *M. abscessus* subsp. bolletii BD genome [6]; two prophages are found in pathogen *M. abscessus* Strain 47J26 [12]; a potential prophage in *M. abscessus* M93 is described [13]; *M. massiliense* Strain M172 contains putative mycobacteriophage [14]; a 55-kb region encodes a putative prophage in *M. canettii* STB-I [15]; a 40-kb prophage is predicted in addition to two prophage-like elements also are seen in *M. simiae* strain DSM 44165 [16]. Many *Mycobacterium* prophages remain to be characterized. Knowledge regarding their genomic composition, distribution can facilitate the elucidation of the biology of *Mycobacterium*.

In this study, we screened all available *Mycobacterium* complete genomes sequences from GenBank, shotgun assembly sequences from Whole Genome Shotgun (WGS) databases, and searched for mycobacterioprophages in published literatures. Together, 33 prophages were described in detail, and 11 of them were previously undocumented prophages among *Mycobacterium* genomes. The genomes, gene contents, comparative genomics studies and the relationships among them were characterized.

## Results and discussion

### Prophages in *Mycobacterium*genomes

Though the identification of prophages from sequenced bacterial genomes is difficult [1], prophage sequences can be found by several approaches. Integrases are well-recognized diagnostic markers for prophages within bacterial genomes [17–23]. Web servers and programs for prophages identification are available [24–28]. In this study, we used an integrated protocol to streamline the identification. Firstly, PHAST (PHAge Search Tool) was used to search *Mycobacterium* genomes. Secondly, the presence or absence of the integrase genes was tested to exclude negative results. Finally, mycobacterioprophage sequences were identified based on the homology between prophage ORFs (open reading frames) and known phage genes. Thirty mycobacterial complete genomes (see Additional file 1) were retrieved. Eleven new prophages were identified. The genomic features of these newly identified mycobacterioprophages are described in Table 1.

**Table 1:** **Genomic features of prophages in** ***Mycobacterium*** **genomes**

Prophages	Host	Coordinate	Insertion sites	Size	Putative ***att*** B regions of prophage-like elements	References
phiMAV_1	*M.avium* 104	746,437-794,445	-	48.0 kb	GGACCTGCGGATTAAAAGTC	This study
phiMAV_2		1,446,840-1,463,298	-	16.5 kb	GTGGTCAGCTT	This study
phiMmcs_1	*M.sp.*MCS	3,067,419-3,080,311	tRNA-Pro	12.9 kb	CCGTTGCCGT	This study
phiMmcs_2		4,054,082-4,063,334	-	9.3 kb	GGTGAGGGCGT	This study
phiMkms_1	*M.sp*.KMS	3,085,307-3,098,199	-	12.9 kb	CCGTTGCCGT	This study
phiMkms_2		4,088,564-4,097,816	-	9.3 kb	GGTGAGGGCGT	This study
phiBN42_1	*M.canettii* CIPT 140070010	1,514,379-1,522,784	tRNA-Arg	8.4 kb	GTGCCCCCGGCAGGATTCG	This study
phiBN44_1	*M.canettii* CIPT 140060008	3,433,693-3,444,793	-	11.1 kb	CCGGAGAAGAAGTCATGGTTCT	This study
phiMCAN_1	*M.canettii* CIPT 140010059	1,180,578-1,191,783	-	11.2 kb	GTTCGAGTCCGACTGGGGGCAC	This study
phiMycsm_1	*M.smegmatis* JS623	4,221,770-4,232,715	-	10.9 kb	GCCGACGACG	This study
phiW7S_1	*M.sp*. MOTT36Y	968,527-980,490	tRNA-Ala	12.0 kb	GTTCGCATCGAGTAGGTCAGGGGTTCGATTCCC	This study
phiRv1^a^	*M.tuberculosis* H37Rv	1,779,267-1,788,525	-	9.3 kb	GGTTGGCCGTGG	[8]
phiRv2^a^		2,970,063-2,980,853	tRNA-Val	10.8 kb	CCGCGCAATAAACGCGCAATA	[8]
phiMU01^a^	*M.ulcerans* Agy99	NM	NM	18 kb	NM	[9]
phiMU02^a^		NM	NM	24 kb	NM	[9]
phiMmar01^a^	*M.marinum* M	27,047- 33,095	tRNA-Leu	6.0 kb	NM	[10]
phiMmar02^a^		4,812,334- 4,869,620	tRNA- Lys	57.8 kb	NM	[10]
phiMmar03^a^		5,460,072– 5,470,770	tRNA- Leu	10.7 kb	NM	[10]
phiMmar04^a^		5,628,721– 5,636,467	tRNA- Arg	7.7 kb	NM	[10]
phiMmar05^a^		5,884,651– 5,904,290	tRNA-Phe	19.6 kb	NM	[10]
phiMmar06^a^		2,567,503– 2,589,174	-	21.6 kb	NM	[10]
phiMmar07^a^		3,082,858– 3,100,046	tRNA- Pro	17.2 kb	NM	[10]
phiMmar08^a^		3,808,513-3,851,917	tRNA-Leu	43.0 kb	NM	[10]
phiMmar09^a^		688,611-695,966	tRNA-Gly	7.4 kb	NM	[10]
phiMmar10^a^		4,405,758- 4,430,810	tRNA-Met	25.1 kb	NM	[10]
Prophage Araucaria^a^	*M.abscessus* subsp. bolletii BD Contig17	NM (1,000-65,113)	-	64.1 kb	NM	[6]
phiMAB_1^#^	*M.abscessus* ATCC 19977	1,754,551-1,835,095	tRNA-Met	80.5 kb	NM	[11]
phiMAB_2^#^		233,621-247,981	tRNA-Arg	14.4 kb	NM	[11]
phiMAB_3^#^		770,916-778,753	tRNA-Lys	7.8 kb	NM	[11]
phiMAB_4^#^		4,909,957-4,959,626	-	49.7 kb	NM	[11]
phiMAB47J26_1^#^	*M.abscessus* Strain 47 J26 Contig02	NM (55,744-116,295)	NM (tRNA-Leu)	60.5 kb	NM (GCGGACTTAAAATCCGCCAAGTGTCGGTTCGAGTCCGACTGGGGGCAC)	[12]
phiMAB47j26_2^#^	*M.abscessus* Strain 47 J26 Contig03	NM (58,656-105,841)	-	47.2 kb	NM	[12]
phiOUW_1^#^	*M.abscessus* M93 Contig09	NM (267,663-342,276)	NM (tRNA-Arg)	74.6 kb	NM (GTGCGCCCGAAGGGATTCGAACCCCTAACCTTCTG)	[13]
phiM172_1^#^	*M.massiliense* Strain M172 Contig06	NM (358,576-422,610)	NM (tRNA-Val)	64.0 kb	NM (TTGGTGGGCGCGGAGGGTTTCGAACCC)	[14]
phiM172_2^#^		NM (456,450-513,710)	-	57.3 kb	NM (CAACCAGTCGGCCTGA)	[14]
phiSTB-I_1^#^	*M.canettii* STB-1 (CIPT 140070007)	NM	tRNA-Lys	55 kb	NM	[15]
phiDSM_1^#^	*M.simiae* DSM 44165	NM	NM	40 kb	NM	[16]
phiDSM_2^#^		NM	NM	7 kb	NM	[16]
phiDSM_3^#^		NM	NM	18 kb	NM	[16]

NM means that these data do not be mentioned; parentheses means that these data is shown in this study; - means these prophages are not integrating into tRNA genes; ^a^those prophages has been described and named; ^#^those prophages has been described, but did not be named.

In the WGS databases, some mycobacteria containing prophages are also reported [12–16]. Since the whole genome sequences of these mycobacteria and the specific information of these prophages are not available, we searched for prophages in five mycobacterial shotgun assembly sequences contigs (see Additional file 1) using the method mentioned above. The results showed that prophages were found in some sequences contigs of *M. abscessus* Strain 47J26, *M.abscessus* M93, and *M.massiliense* M172 (Table 1). Prophages previously reported in the genomes of *M.canettii* CIPT 140070007 and *M.simiae* DSM 44165 cannot be detected in our study. With annotated whole genomic sequence, this puzzle might be solved.

Some mycobacteria harboring prophages have been detailed in previous studies [6, 8, 10, 11], which are included in Table 1. Four of them contained in *M.abscessus* ATCC 19977 chromosome are not designated. We named them phiMAB_1, phiMAB_2, phiMAB_3, and phiMAB_4, respectively. We noted that two prophage, PhiMU01 and PhiMU02, mentioned in *M.ulcerans* Agy99 genome, lack specific information and cannot be detected.

Overall, thirty-three prophages were described, and six prophages had been mentioned, but without specific information. Eleven prophages were found from the complete genome database; five prophages were retrieved from the WGS databases; seventeen of them were reported prophages with specific sequence information. Their size range was from 6 kb to 80.5 kb. Based on the length of prophage genome (the length of mycobacteriophage genomes is 41,441 bp – 164,602 bp, http://phagesdb.org/), 11 prophages can be considered as full-length prophage. The remaining 22 prophages were prophage-like elements. The result showed that small prophage-like elements were more prevalent than putative full-length prophages. The small prophage-like elements might be more stable due to mutational decay and loss of some genes somehow involved in genome excision. Small prophage-like elements were more stable and can be more easily detected than the full-length prophages. Through the tRNA search tool, 19 prophages were integrated into tRNA genes (Table 1). The frequency of tRNA integration was tRNA-Leu (4/19), tRNA-Arg (4/19), tRNA-Val (2/19), tRNA-Lys (2/19), tRNA-Pro (2/19), tRNA-Met (2/19), tRNA-Phe (1/19), tRNA-Gly (1/19), tRNA-Ala (1/19). The genome of *M.*sp.KMS, *M.*sp.MCS, *M.avium* 104, *M.tuberculosis* H37Rv, *M.marinum* M, *M.abscessus* ATCC 19977, *M.abscessus* Strain 47J26, and *M.massiliense* Strain M172 was polylysogenic.

### New prophages of *Mycobacterium*genomes

#### Full-length prophage phiMAV_1 in the genome of M. avium 104

Prophage phiMAV_1, spanning from MAV_0779 (integrase gene) to MAV_0841 (excisionase DNA binding protein), contains sixty-three ORFs (see Additional file 2), and is flanked by two 20-bp repeats (Table 1) reminiscent of *att*L and *att*R sites. There is no predicted tRNA within the prophage. PhiMAV_1 cannot be categorized into any known phage clusters and might represent new singleton type [29].

**Figure 1:**
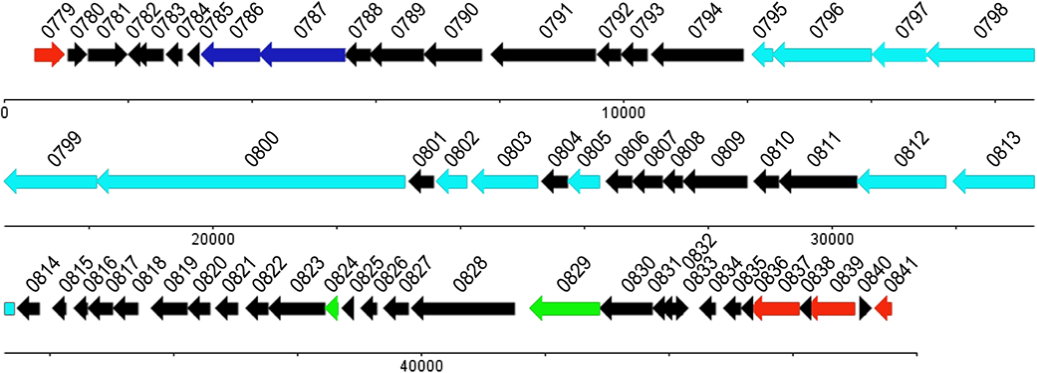
**The genomic organization of** ***M.avium*** **104 full-length prophage phiMAV_1.** The red arrows represent lysogeny module; the blue arrows represent lysis module; the cyan arrows represent DNA packaging and structural modules; the green arrows represent DNA metabolism module. Numbers means the numbering of gene.

Based on Blast-p, 41 phiMAV_1 ORFs show more or less amino acid sequence similarity to other known phage genes, and 17 can be assigned functionalities based on homology (see Additional file 2). PhiMAV_1 genome consists of different functional modules (Figure 1).

The lysis module consists of MAV_0786 and MAV_0787, which encode cutinase and glycosyl hydrolase respectively that can lyze bacterium and enable the release of progeny phages. The DNA packaging and structural modules extend from MAV_0795 to MAV_0813. MAV_0795, MAV_0797, and MAV_0803 all encode putative tail protein. MAV_0798 and MAV_0799 all encode putative structural protein. MAV_0800, MAV_0802, and MAV_0805 encode phage tail tape measure protein, tail assembly chaperone, and phage capsid and scaffold protein. MAV_0812 and MAV_0813 encode putative portal protein and phage terminase engaged in the phage head morphogenesis. The DNA metabolism module includes MAV_0824 and MAV_0829. MAV_0824 encodes exonuclease and MAV_0829 encodes recombination and repair protein RecT. The lysogeny module consists of MAV_0837, MAV_0839, MAV_0841 and MAV_0779. MAV_0779 and MAV_0841 encode phage integrase and excisionase DNA binding protein. Both MAV_0837 and MAV_0839 encode phage antirepressor protein.

In addition to ORFs similar to other phage genes, two ORFs show unexpected similarity to bacterial key proteins. MAV_0835 encodes type VI secretion protein IcmF (Intracellular Multiplication F), a core component of type VI secretion system in *Pseudomonas aeruginosa*, *Vibrio cholerae* or other pathogenic bacteria [30–32]. Based on Blast-p, type VI secretion system was not documented in mycobacteria except for *M.avium* 104 and *M.parascrofulaceum.* IcmF is involved in bacterial motility, adherence to epithelial cells, and conjugation frequency [31], and has been reported in an avian pathogenic *Escherichia coli* (APEC) strain [32]. In addition, MAV_0790 encodes PPE family protein, a widespread *Mycobacterium* unique protein. This implies that MAV_0835 and MAV_0790 play a role in the physiology and pathogenicity of *M.avium* 104.

#### Prophage-like elements phiMAV_2

**Figure 2:**
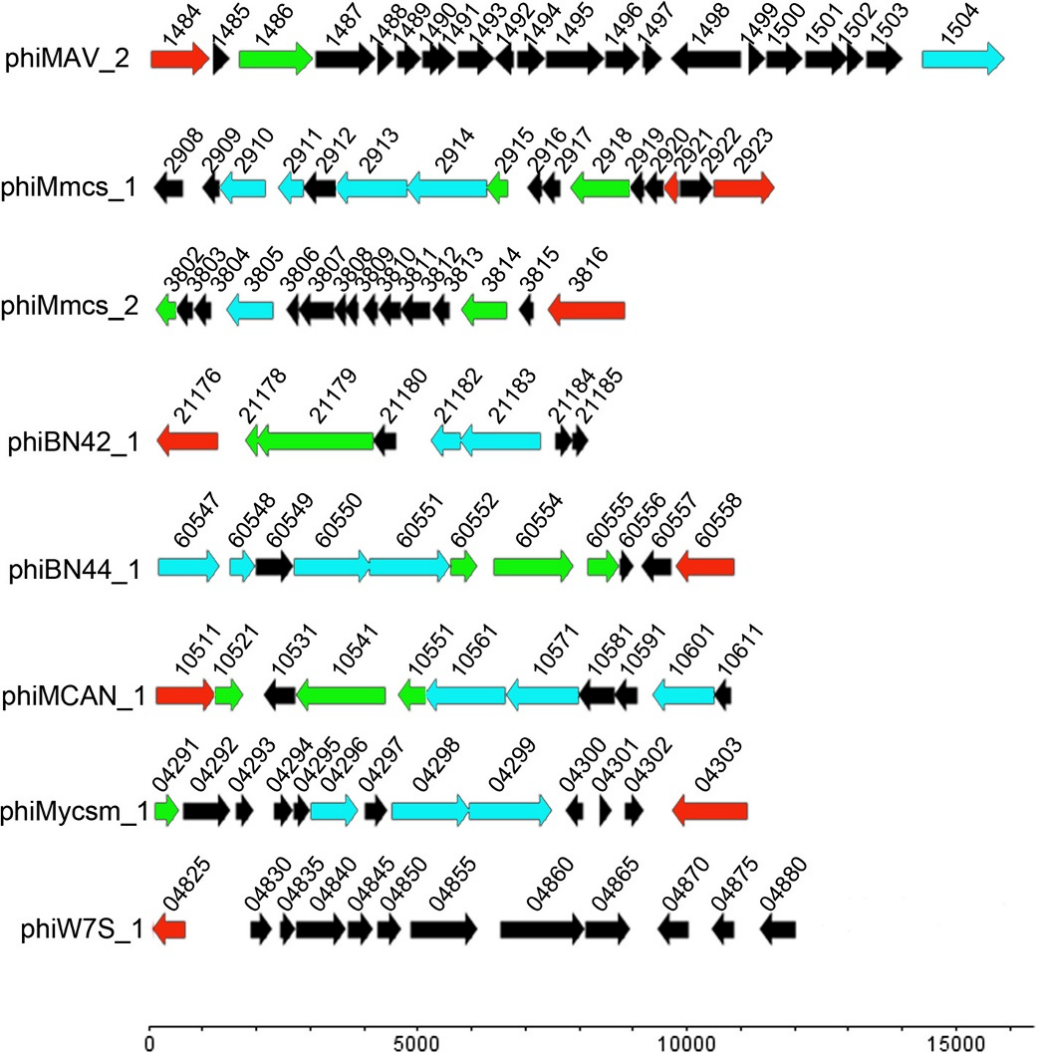
**Genomic organization of some defective prophage-like elements among mycobacteria.** Numbers means the numbering of gene. The red arrows represent lysogeny module; the blue arrows represent lysis module; the cyan arrows represent DNA packaging and structural modules; the green arrows represent DNA metabolism module.

Prophage phiMAV_2 (Figure 2), integrated into a hypothetical gene (MAV_1505) in *M.avium* 104, extends from MAV_1484 (integrase gene) to MAV_1504 (Phage terminase) and contains 21 ORFs (see Additional file 3) flanked by an 11-bp repeat (Table 1), indicative of *att*L and *att*R sites. No tRNA is found in the genome of phiMAV_2. Based on Blast-p, only nine ORFs have sequence similarity to other phage genes at the amino acid sequence level. Six ORFs of the phiMAV_2 prophage genome can be assigned function based on database search, namely the integrase gene (MAV_1484), response regulator receiver protein (MAV_1485), DNA primase/polymerase (MAV_1486), Y4cG protein (MAV_1493), transposase (MAV_1498) and phage terminase (MAV_1504). Other phiMAV_2 prophage ORFs similar to known bacterial functional proteins are also identified (see Additional file 3).

#### Prophage-like elements phiMmcs_1, phiMmcs_2, phiMkms_1, and phiMkms_2

There are two prophage-like elements in *M.sp.*MCS, phiMmcs_1 and phiMmcs_2. Prophage phiMmcs_1 (Figure 2), which is integrated into a tRNA-pro (Mmcs_R0021) in *M.sp.*MCS, extends from Mmcs_2923 (integrase gene) to Mmcs_2908 (transglycosylase-like protein) and contains sixteen ORFs (see Additional file 4) flanked by a 10-bp repeat (Table 1), indicative of *att*L and *att*R sites. No tRNA is found in the genome of phiMmcs_1. Only nine ORFs can be assigned function based on amino acid sequence homology. The prophage phiMmcs_1 genome contains 4 modules. The lysis module appeared to be limited to Mmcs_2908, whose protein product has 50% sequence identity to lysin of *Rhodococcus phage* REQ1. The structural module consists of Mmcs_2910 and Mmcs_2914. Mmcs_2910, Mmcs_2911, Mmcs_2913, and Mmcs_2914 encode phage major capsid protein, scaffolding protein, phage portal protein, and phage terminase, respectively. The DNA metabolism module has two genes (Mmcs_2915 and Mmcs_2918), whose predicted protein products are HNH endonuclease and DNA repair protein RadA, respectively. The lysogeny module consists of Mmcs_2921 (putative phage excisionase) and Mmcs_2923 (phage integrase).

The phiMmcs_2 prophage remnant inserts between Mmcs_3803 and Mmcs_3817. The prophage sequence contains 15 ORFs (see Additional file 5) and is flanked by two 11-bp repeats, indicating the existence of putative *att*L and *att*R sites. Based on Blast-p, only 8 ORFs have sequence similarity to other phage genes at the amino acid sequence level and 4 can be assigned function, namely Mmcs_3802 (HNH endonuclease), Mmcs_3805 (phage major capsid protein), Mmcs_3814 (HNH endonuclease domain-containing protein), and Mmcs_3816 (phiRv1 integrase).

PhiMkms_1 and phiMkms_2 (see Additional files 6 and 7) are prophage-like elements in *M.sp*.KMS. PhiMmcs_1 is identical to phiMkms_1 and represents same prophage. They also insert into the same location in host genome. PhiMmcs_2 and phiMkms_2 is just the same scenario as phiMkms_1 and phiMkms_2.

#### Prophage-like elements phiBN42_1, phiBN44_1, and phiMCAN_1

PhiBN42_1, phiBN44_1, and phiMCAN_1 are found in *M. canettii* CIPT 140070010, *M.canettii* CIPT 140060008, and *M.canettii* CIPT 140010059 respectively. Prophage phiBN42_1 (Figure 2), which is integrated into a tRNA-arg (BN42_tRNA41) in *M.canettii* CIPT 140070010, extends from BN42_21176 (integrase gene) to BN42_21185 (hypothetical protein) and contains only eight ORFs (see Additional file 8) flanked by a 19-bp repeat (Table 1), indicative of *att*L and *att*R sites. No tRNA is found in the genome of phiBN42_1. Only seven genes have sequence similarity to other phage genes, five of which can be assigned function. There are BN42_21176 (integrase), BN42_21178 (excisionase), BN42_21179 (DNA primase), BN42_21182 (phage prohead protease), and BN42_21183 (phage major capsid protein).

The phiBN44_1 prophage remnant is located between BN44_60546 and BN44_60559 in *M.canettii* CIPT 140060008, flanked by a 22-bp repeat (Table 1), representing candidates for the *att*L and *att*R sites. There are 11 ORFs in phiBN44_1 prophage genome (see Additional file 9). Eight are similar to other phage genes and can be assign function. There are BN44_60547 (phage major capsid protein), BN44_60548 (scaffolding protein), BN44_60550 (Phage portal protein), BN44_60551 (Phage Terminase), BN44_60552 (HNH endonuclease), BN44_60554 (DNA primase), BN44_60557 (XRE family transcriptional regulator), and BN44_60558 (phage integrase). Additionally, BN44_60555 encodes protein similar to *Human adenovirus* DNA polymerase and BN44_60556 encodes protein similar to K^+^ transporter of many bacteria.

Prophage phiMCAN_1 (Figure 2), which is integrated into between MCAN_10501 and MCAN_10621 in *M.canettii* CIPT 140010059, contains only 11 ORFs flanked (see Additional file 10) by a 22-bp repeat (Table 1), indicative of *att*L and *att*R sites. No tRNA is found in the genome of phiMCAN_1. Only 8 ORFs similar to other phage genes at the amino acid sequence level and seven genes have been assigned function. There are MCAN_10511 (phage integrase), MCAN_10521 (DNA-binding protein), MCAN_10541 (DNA primase), MCAN_10551 (HNH endonuclease), MCAN_10561 (phage terminase), MCAN_10571 (phage portal protein), and MCAN_10601 (phage major capsid protein).

#### Prophage-like elements phiMycsm_1 and phiW7S_1

Prophage phiMycsm_1 (Figure 2), inserted between Mycsm_04290 and Mycsm_04304 in *M.smegmatis* JS623, contains 13 ORFs (see Additional file 11) flanked by a 10-bp repeat (Table 1), indicative of *att*L and *att*R sites. No tRNA is found in the genome of phiMycsm_1. Nine ORFs show the protein sequence similarity to other phage genes, in which six ORFs have the descriptive function: Mycsm_04291 (phage integrase), Mycsm_04296 (DNA-binding protein), Mycsm_04298 (DNA primase), Mycsm_04299 (HNH endonuclease), Mycsm_04302 (phage terminase), and Mycsm_04303 (phage portal protein). Additionally, Mycsm_04293, whose protein product is similar to glycerate kinase, is also present in phiBN44_1.

Prophage phiW7S_1 (Figure 2) integrated into a tRNA-ala (W7S_t25871) in *M.sp*. MOTT36Y, extends from W7S_04825 (integrase gene) to W7S_04880 (hypothetical protein) and contains 12 ORFs (see Additional file 12) flanked by a 33-bp repeat (Table 1), indicative of *att*L and *att*R sites. No tRNA is found in the genome of phiW7S_1. Only six genes have sequence similarity to other phage genes and three of them have annotated function, which are W7S_04825 (integrase), W7S_04845 (pantothenate kinase), and W7S_04855 (transposase).

### Grouping of full-length prophages

We searched all the literatures published so far about full-length mycobacterioprophages. Only one prophage Araucaria is assigned to a Dori-like prophage [6]. BlastN (http://phagesdb.org/blast/) and dot plot matrix of the genomes of full-length mycobacterioprophages and mycobacteriophage clusters (A-T and singletons) revealed that phi172_2 shared sequence similarity to cluster A (see Additional file 13); phiMAB_1 shared an even weaker sequence similarity to subcluster F1 (see Additional file 14); phiMAB47J26_1 shared an even weak sequence similarity to subcluster F1 and cluster N (see Additional file 15); phiMAB47J26_2 shared an even weak sequence similarity to cluster P, subcluster F1, and cluster N (see Additional file 16); phi172_1 shared an even weaker sequence similarity to subcluster F1 and cluster N (see Additional file 17). The remaining full-length prophages had no close relatives to any cluster. We proposed that phi172_2 was grouped into cluster A, and other full-length mycobacterioprophages did not belong to any mycobacteriophage clusters and were ‘singletons’.

### Comparative genomics of prophage-like elements

**Figure 3:**
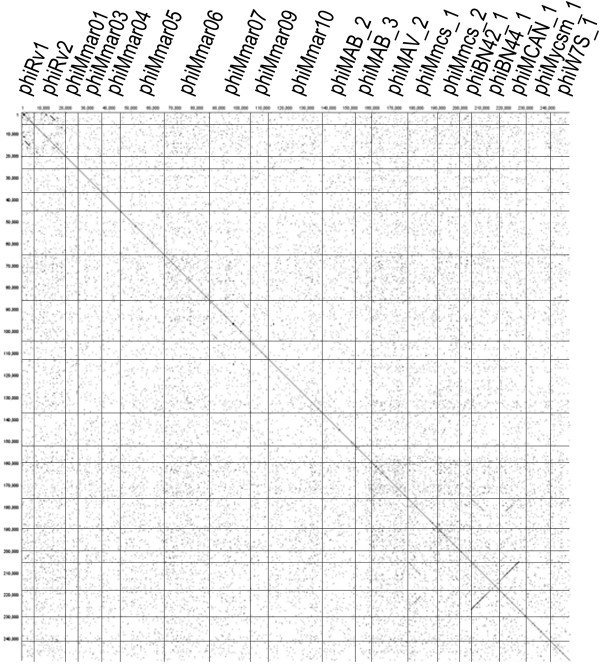
**Comparative genomic analyses of prophage-like sequences.** Dot plot matrix calculated for the complete genomes of all prophage-like sequences in *Mycobacterium*. The top x axis and the left y axis provide a scale in kilobases; and the top x axis identifies the prophage genomes that are compared in the corresponding square. The x and y axes are the identical sequences. The slash means that two DNA fragments are homologous to each other. The backslash means that one DNA fragment is homologous with the reverse sequence of other DNA fragment. The word length used is 12 bp.

**Figure 4:**
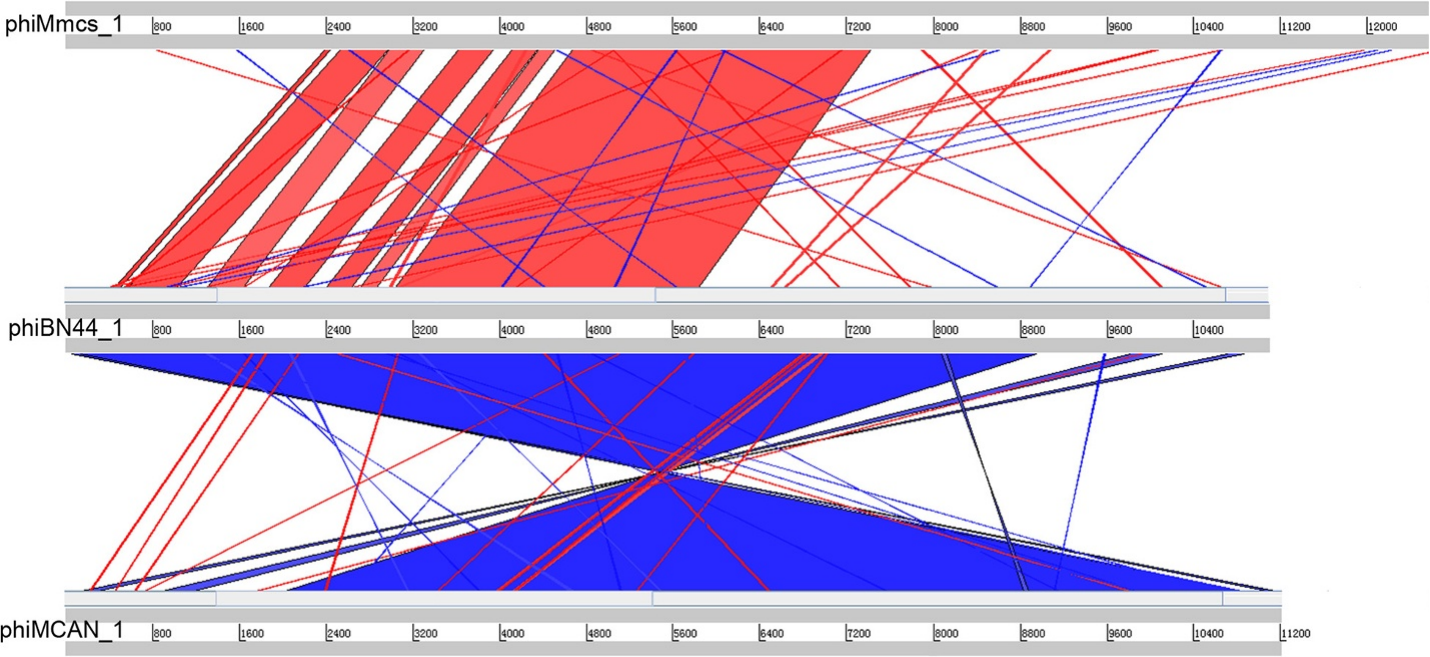
**Global comparison of phiMmcs_1 (or phiMkms_1), phiBN44_1, and phiMCAN_1.** Highly related sequences are shown by the red shadings. The blue shadings means that the DNA fragments are highly homologous to complementary sequence of other fragments.

Dot plot matrix was generated for the complete genomes of 22 mycobacterioprophage-like elements in this study (Figure 3). The figure displays that phiMmcs_1, phiMkms_1, phiBN44_1, and phiMCAN_1 are more closely related to each other than to other mycobacterioprophage-like elements, and can be classified as one group. In a simple NCBI ‘Align two sequences’comparison, the comparison between phiMmcs_1 (or phiMkms_1) and phiBN44_1 shows that one of the major segments less than 2801 bp has greater than 71% identity, and four segments less than 200 bp are reported to have 68% identity (Figure 4). The comparison between reverse complementary sequence of phiMCAN_1 and phiBN44_1 shows that one of the major segments 8952 bp has greater than 85% identity (Figure 4). Further analysis indicated a lack of homology between the prophage of *M.tuberculosis* H37Rv and other prophage-like elements.

### Phylogeny of prophage integrases

**Figure 5:**
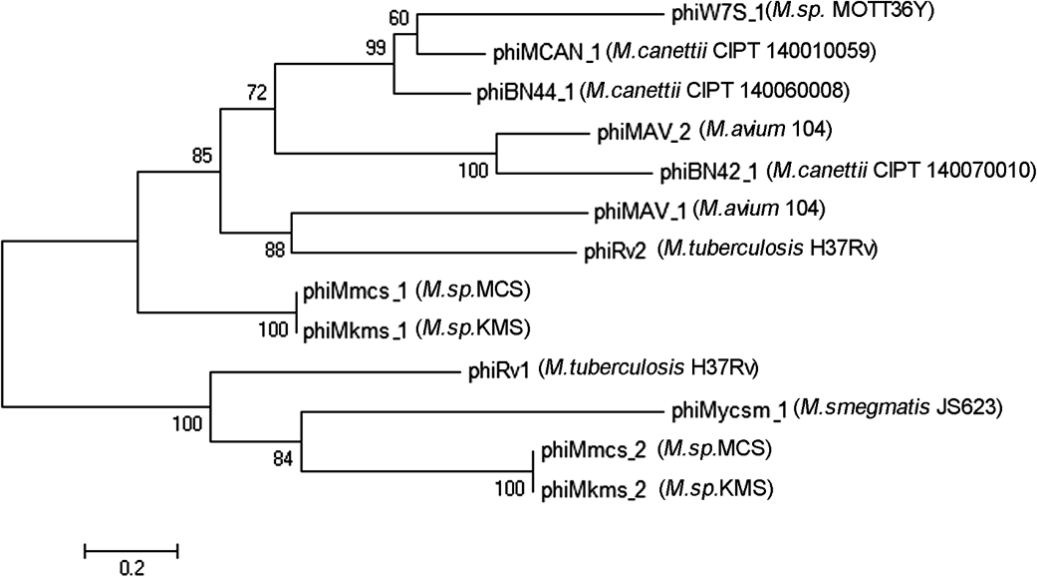
**Phylogeny of prophage integrases.** Unrooted phylogenetic relationships are represented using NJTree. Bootstrap values from 1,000 reiterations are shown.

Integrase can be found in virtually each prophage genome found in this study. And it can serve as good marker for the phylogeny of prophage phiRv1 element encodes a serine site-specific recombinase and phiRv2 encodes a tyrosine recombinase [33]. All integrases fall into the two categories (Figure 5). The serine recombinase division includes phiMycsm_1, phiMmcs_2 (phiMkms_2) and phiRv1. The tyrosine recombinase division includes the remaining prophages and phiRv2. PhiMmcs_1 (phiMkms_1), phiBN44_1, and phiMCAN_1 belong to the same clade, consistent with the comparative genomic result. The distance between prophages had little relevance to the phylogeny between their hosts, suggestive of independent evolutionary trajectory.

## Conclusions

In brief, we present here thirty-three mycobacterioprophages mined from sequenced mycobacterial genomes, the WGS databases, and some published literatures. Eleven prophages were newly identified prophages from complete genome database; five prophages were from the WGS databases; seventeen prophages were reported with specific sequence information. The genome sequences, gene contents of eleven newly identified prophages were analyzed. Comparative genomic analysis revealed that one full-length mycobacterioprophage phi172_2 belonged to cluster A and one group having recognizable sequence similarity was verified and contained four small prophage-like elements, including the phiMmcs_1, phiMkms_1, phiBN44_1, and phiMCAN_1. To our knowledge, this represents the first systematic analysis of mycobacterioprophages. With more forthcoming *Mycobacterium* genome sequences and thorough mycobacterioprophages screening, we can generate a more comprehensive picture of the role of prophages in mycobacterial evolution, adaptations and physiology.

## Methods

### Data collection and mycobacterioprophage identification

DNA sequences of bacteria for analysis were downloaded from multiple databases, such as NCBI (the National Center for Biotechnology Information). PHAST (http://phast.wishartlab.com/index.html) were firstly used for analyzing bacterial genome to find candidate prophages [24]. An integrase gene was screened from candidate prophage genome for in these results to drop false negative results [17–20]. Finally, prophages were identified on the basis of the presence of significant homology between ORFs (open reading frames) and known phage genes [17].

### Analysis of mycobacterioprophage genome sequence

Prophage sequence was annotated using a variety of programs including Glimmer [34]. tRNA and tmRNA genes were identified using tRNA-Scan-SE (http://lowelab.ucsc.edu/tRNAscan-SE/) [35] and ARAGORN (http://mbio-serv2.mbioekol.lu.se/ARAGORN/) [36]. BLAST analyses were performed remotely at the NCBI (http://blast.ncbi.nlm.nih.gov/Blast.cgi) and the phagesdb.org site (http://phagesdb.org/blast/). Some data about mycobacteriophage genomes was downloaded from the phagesdb.org site (http://phagesdb.org/). DNAman was used to searching the flank of prophage to find *att*L and *att*R sites. Sequences were submitted entries to the GenBank sequence database by Sequin (http://www.ncbi.nlm.nih.gov/projects/Sequin/index.html). Comparative genomic analyses of prophage could be carried out by Blast-N for the global comparison of phiMmcs_1 (or phiMkms_1), phiBN44_1, and phiMCAN_1 and Geneious software for the dotplot of all the mycobacterioprophage-like sequences [37]. Multiple sequence alignment and the construct of phylogenetic trees were performed using ClustalW (http://embnet.vital-it.ch/software/ClustalW.html) or MEGA4 [38].

## Electronic supplementary material

Additional file 1: Table S1: Mycobacterial genomes retrieved in this study. (DOC 56 KB)

Additional file 2: Table S2: Database matches for phiMAV_1. (DOC 85 KB)

Additional file 3: Table S3: Database matches for phiMAV_2. (DOC 44 KB)

Additional file 4: Table S4: Database matches for phiMmcs_1. (DOC 40 KB)

Additional file 5: Table S5: Database matches for phiMmcs_2. (DOC 38 KB)

Additional file 6: Table S6: Database matches for phiMkms_1. (DOC 40 KB)

Additional file 7: Table S7: Database matches for phiMkms_2. (DOC 39 KB)

Additional file 8: Table S8: Database matches for phiBN42_1. (DOC 30 KB)

Additional file 9: Table S9: Database matches for phiBN44_1. (DOC 34 KB)

Additional file 10: Table S10: Database matches for phiMCAN_1. (DOC 33 KB)

Additional file 11: Table S11: Ddatabase matches for phiMycsm_1. (DOC 34 KB)

Additional file 12: Table S12: Database matches for phiW7S_1. (DOC 34 KB)

Additional file 13: Figure S1-S11: Comparative genomic analyses of phi172_2 and cluster A (subcluster A1-A11) mycobacteriophage. (DOC 5 MB)

Additional file 14: Figure S12: Comparative genomic analyses of phiMAB_1 and subcluster F1 mycobacteriophage. (DOC 549 KB)

Additional file 15: Figure S13-S14: Comparative genomic analyses of phiMAB47J26_1, subcluster F1 and cluster N mycobacteriophage. (DOC 1 MB)

Additional file 16: Figure S15-S17: Comparative genomic analyses of phiMAB47J26_2, cluster P, subcluster F1 and cluster N mycobacteriophage. (DOC 2 MB)

Additional file 17: Figure S18-S19: Comparative genomic analyses of phi172_1, subcluster F1 and cluster N mycobacteriophage. (DOC 1 MB)
