# Supplementary material for: Prophage-like elements present in Mycobacterium genomes
Source: BMC Genomics. 2014 Mar 27;15(1):243. doi: 10.1186/1471-2164-15-243 (PMC3986857; doi:10.1186/1471-2164-15-243)
Supplement: Supplementary file 1 — Additional file 1: Table S1: Mycobacterial genomes retrieved in this study. (DOC 56 KB) [file 12864_2013_7046_MOESM1_ESM.doc]

Table S1 Mycobacterial genomes retrieved in this study.

| Host | Genbank accession number | Prophage |
| --- | --- | --- |
| *Mycobacterium*massiliense str. GO 06 | NC_018150 | - |
| *Mycobacterium avium* 104 | NC_008595 | phiMAV_1 and phiMAV_2 |
| *Mycobacterium* avium subsp. paratuberculosis K-10 | NC_002944 | - |
| *Mycobacterium* sp. JDM601 | NC_015576 | - |
| *Mycobacterium* sp. JLS | NC_009077 | - |
| *Mycobacterium* sp. KMS | NC_008705 | phiMkms_1 and phiMkms_2 |
| *Mycobacterium* sp. MCS | NC_008146 | phiMmcs_1 and phiMmcs_2 |
| *Mycobacterium* sp. MOTT36Y | NC_017904 | phiW7S_1 |
| *Mycobacterium* *canettii* CIPT 140070017 | NC_019952 | - |
| *Mycobacterium* *canettii* CIPT 140010059 | NC_015848 | phiMCAN_1 |
| *Mycobacterium* *canettii* CIPT 140060008 | NC_019950 | phiBN44_1 |
| *Mycobacterium* *canettii* CIPT 140070008 | NC_019965 | - |
| *Mycobacterium* *canettii* CIPT 140070010 | NC_019951 | phiBN42_1 |
| *Mycobacterium* *intracellulare* MOTT-64 | NC_016948 | - |
| *Mycobacterium* *intracellulare* ATCC 13950 | NC_016946 | - |
| *Mycobacterium* *intracellulare* MOTT-02 | NC_016947 | - |
| *Mycobacterium* *leprae* TN | NC_002677 | - |
| *Mycobacterium* *leprae* Br4923 | NC_011896 | - |
| *Mycobacterium* *kansasii* ATCC 12478 | NZ_CM000636 | - |
| *Mycobacterium* *smegmatis* JS623 | NC_019966 | phiMycsm_1 |
| *Mycobacterium* *smegmatis* str. MC^2^ 155 | NC_008596 | - |
| *Mycobacterium* *smegmatis* str. MC2 155 | NC_018289 | - |
| *Mycobacterium gilvum* PYR-GCK | NC_009338 | - |
| *Mycobacterium gilvum* Spyr1 | NC_014814 | - |
| *Mycobacterium rhodesiae* NBB3 | NC_016604 | - |
| *Mycobacterium* *liflandii* 128FXT | NC_020133 | - |
| *Mycobacterium* *indicus* pranii | NC_018612 | - |
| *Mycobacterium* *chubuense* NBB4 | NC_018027 | - |
| *Mycobacterium* *vanbaalenii* PYR-1 | NC_008726 | - |
| *Mycobacterium africanum* GM041182 | NC_015758 | - |
| *Mycobacterium abscessus* Strain 47J26 | AGQU00000000 | phiMAB47J26_1 and phiMAB47J26_1 |
| *Mycobacterium abscessus* M93 | AJGF00000000 | phiOUW_1 |
| *Mycobacterium massiliense* Strain M172 | AJSE00000000 | phiM172_1 and phiM172_1 |
| *Mycobacterium canettii* STB-1 (CIPT 140070007) | CAOO00000000 | - |
| *Mycobacterium simiae* DSM 44165 | CBMJ000000000 | - |

‘-’ means that prophage is not found.
