# Supplementary material for: Prophage-like elements present in Mycobacterium genomes
Source: BMC Genomics. 2014 Mar 27;15(1):243. doi: 10.1186/1471-2164-15-243 (PMC3986857; doi:10.1186/1471-2164-15-243)
Supplement: Supplementary file 2 — Additional file 2: Table S2: Database matches for phiMAV_1. (DOC 85 KB) [file 12864_2013_7046_MOESM2_ESM.doc]

Table S2 Database matches for phiMAV_1

| Gene | Function | Whether it is similar to phage protein |
| --- | --- | --- |
| MAV_0779 | phage integrase | yes |
| MAV_0780 | hypothetical protein | yes |
| MAV_0781 | hypothetical protein | no |
| MAV_0782 | hypothetical protein | no |
| MAV_0783 | hypothetical protein | yes |
| MAV_0784 | hypothetical protein | no |
| MAV_0785 | hypothetical protein | no |
| MAV_0786 | cutinase | yes |
| MAV_0787 | glycosyl hydrolase family 25 | yes |
| MAV_0788 | hypothetical protein | no |
| MAV_0789 | hypothetical protein | no |
| MAV_0790 | PPE family protein | no |
| MAV_0791 | hypothetical protein | yes |
| MAV_0792 | hypothetical protein | yes |
| MAV_0793 | hypothetical protein | yes |
| MAV_0794 | hypothetical protein | no |
| MAV_0795 | major tail subunit | yes |
| MAV_0796 | hypothetical protein | yes |
| MAV_0797 | putative tail protein | yes |
| MAV_0798 | putative structural protein | yes |
| MAV_0799 | putative structural protein | yes |
| MAV_0800 | phage tail tape measure protein | yes |
| MAV_0801 | hypothetical protein | no |
| MAV_0802 | tail assembly chaperone | yes |
| MAV_0803 | putative major tail unit | yes |
| MAV_0804 | hypothetical protein | yes |
| MAV_0805 | Phage capsid and scaffold protein | yes |
| MAV_0806 | hypothetical protein | no |
| MAV_0807 | hypothetical protein | yes |
| MAV_0808 | hypothetical protein | yes |
| MAV_0809 | hypothetical protein | yes |
| MAV_0810 | hypothetical protein | yes |
| MAV_0811 | hypothetical protein | yes |
| MAV_0812 | putative portal protein | yes |
| MAV_0813 | phage terminase, large subunit | yes |
| MAV_0814 | hypothetical protein | yes |
| MAV_0815 | hypothetical protein | yes |
| MAV_0816 | hypothetical protein | no |
| MAV_0817 | hypothetical protein | yes |
| MAV_0818 | hypothetical protein | yes |
| MAV_0819 | hypothetical protein | yes |
| MAV_0820 | hypothetical protein | no |
| MAV_0821 | hypothetical protein | no |
| MAV_0822 | hypothetical protein | yes |
| MAV_0823 | hypothetical protein | yes |
| MAV_0824 | excinuclease ABC subunit C | yes |
| MAV_0825 | hypothetical protein | no |
| MAV_0826 | hypothetical protein | yes |
| MAV_0827 | hypothetical protein | no |
| MAV_0828 | 17 kDa surface antigen family protein | no |
| MAV_0829 | recombination and repair protein RecT | yes |
| MAV_0830 | hypothetical protein | yes |
| MAV_0831 | hypothetical protein | yes |
| MAV_0832 | hypothetical protein | yes |
| MAV_0833 | hypothetical protein | no |
| MAV_0834 | hypothetical protein | yes |
| MAV_0835 | type VI secretion protein IcmF/ intracellular multiplication and macrophage-killing family protein | no |
| MAV_0836 | hypothetical protein | no |
| MAV_0837 | phage antirepressor protein | yes |
| MAV_0838 | hypothetical protein | no |
| MAV_0839 | phage antirepressor protein | yes |
| MAV_0840 | hypothetical protein | no |
| MAV_0841 | excisionase DNA binding protein | no |
