# Supplementary material for: Prophage-like elements present in Mycobacterium genomes
Source: BMC Genomics. 2014 Mar 27;15(1):243. doi: 10.1186/1471-2164-15-243 (PMC3986857; doi:10.1186/1471-2164-15-243)
Supplement: Supplementary file 3 — Additional file 3: Table S3: Database matches for phiMAV_2. (DOC 44 KB) [file 12864_2013_7046_MOESM3_ESM.doc]

Table S3 Database matches for phiMAV_2

| gene | function | Whether it is similar to phage protein |
| --- | --- | --- |
| MAV_1484 | integrase | yes |
| MAV_1485 | response regulator receiver protein | yes |
| MAV_1486 | DNA primase/polymerase domain protein | yes |
| MAV_1487 | hypothetical protein | yes |
| MAV_1488 | hypothetical protein | no |
| MAV_1489 | hypothetical protein | yes |
| MAV_1490 | flagellar hook-length control protein | no |
| MAV_1491 | hypothetical protein | no |
| MAV_1493 | Y4cG protein/ integrase | yes |
| MAV_1492 | transcriptional regulator | no |
| MAV_1494 | cadmium inducible protein cadi | no |
| MAV_1495 | arsenical-resistance protein | no |
| MAV_1496 | low molecular weight phosphotyrosine protein phosphatase | no |
| MAV_1497 | arsenate reductase | no |
| MAV_1498 | transposase, Mutator family protein | yes |
| MAV_1499 | hypothetical protein | no |
| MAV_1500 | hypothetical protein | no |
| MAV_1501 | hypothetical protein | yes |
| MAV_1502 | thioredoxin | no |
| MAV_1503 | Mannose-specific phosphotransferase enzyme IIB | no |
| MAV_1504 | Phage terminase, ATPase subunit | yes |
