# Supplementary material for: Prophage-like elements present in Mycobacterium genomes
Source: BMC Genomics. 2014 Mar 27;15(1):243. doi: 10.1186/1471-2164-15-243 (PMC3986857; doi:10.1186/1471-2164-15-243)
Supplement: Supplementary file 4 — Additional file 4: Table S4: Database matches for phiMmcs_1. (DOC 40 KB) [file 12864_2013_7046_MOESM4_ESM.doc]

Table S4 Database matches for phiMmcs_1

| gene | function | Whether it is similar to phage protein |
| --- | --- | --- |
| Mmcs_2906 | periplasmic sensor signal transduction histidine kinase | no |
| Mmcs_2907 | serine/threonine protein kinase | no |
| Mmcs_2908 | transglycosylase-like protein | yes |
| Mmcs_2909 | hypothetical protein | no |
| Mmcs_2910 | phage major capsid protein | yes |
| Mmcs_2911 | scaffolding protein | yes |
| Mmcs_2912 | acetylornithine deacetylase | no |
| Mmcs_2913 | Phage portal protein | yes |
| Mmcs_2914 | phage terminase | yes |
| Mmcs_2915 | HNH endonuclease | yes |
| Mmcs_2916 | hypothetical protein | no |
| Mmcs_2917 | hypothetical protein | no |
| Mmcs_2918 | DNA repair protein RadA | yes |
| Mmcs_2919 | hypothetical protein | no |
| Mmcs_2920 | hypothetical protein | no |
| Mmcs_2921 | putative phage excisionase | yes |
| Mmcs_2922 | hypothetical protein | no |
| Mmcs_2923 | phage integrase | yes |
