# Supplementary material for: Prophage-like elements present in Mycobacterium genomes
Source: BMC Genomics. 2014 Mar 27;15(1):243. doi: 10.1186/1471-2164-15-243 (PMC3986857; doi:10.1186/1471-2164-15-243)
Supplement: Supplementary file 5 — Additional file 5: Table S5: Database matches for phiMmcs_2. (DOC 38 KB) [file 12864_2013_7046_MOESM5_ESM.doc]

Table S5 Database matches for phiMmcs_2

| gene | function | Whether it is similar to phage protein |
| --- | --- | --- |
| Mmcs_3802 | HNH endonuclease | yes |
| Mmcs_3803 | hypothetical protein | no |
| Mmcs_3804 | hypothetical protein | yes |
| Mmcs_3805 | phage major capsid protein | yes |
| Mmcs_3806 | hypothetical protein | no |
| Mmcs_3807 | hypothetical protein | yes |
| Mmcs_3808 | hypothetical protein | no |
| Mmcs_3809 | hypothetical protein | no |
| Mmcs_3810 | methylmalonyl-CoA mutase | no |
| Mmcs_3811 | hypothetical protein | yes |
| Mmcs_3812 | excinuclease ABC subunit C | no |
| Mmcs_3813 | hypothetical protein | yes |
| Mmcs_3814 | HNH endonuclease domain-containing protein | yes |
| Mmcs_3815 | hypothetical protein | no |
| Mmcs_3816 | PhiRv1 integrase | yes |
