# Supplementary material for: Prophage-like elements present in Mycobacterium genomes
Source: BMC Genomics. 2014 Mar 27;15(1):243. doi: 10.1186/1471-2164-15-243 (PMC3986857; doi:10.1186/1471-2164-15-243)
Supplement: Supplementary file 6 — Additional file 6: Table S6: Database matches for phiMkms_1. (DOC 40 KB) [file 12864_2013_7046_MOESM6_ESM.doc]

Table S6 Database matches for phiMkms_1

| gene | function | Whether it is similar to phage protein |
| --- | --- | --- |
| Mkms_2950 | periplasmic sensor signal transduction histidine kinase | no |
| Mkms_2951 | serine/threonine protein kinase | no |
| Mkms_2952 | transglycosylase-like protein | yes |
| Mkms_2953 | hypothetical protein | no |
| Mkms_2954 | phage major capsid protein | yes |
| Mkms_2955 | scaffolding protein | yes |
| Mkms_2956 | acetylornithine deacetylase | no |
| Mkms_2957 | Phage portal protein | yes |
| Mkms_2958 | phage terminase | yes |
| Mkms_2959 | HNH endonuclease | yes |
| Mkms_2960 | hypothetical protein | no |
| Mkms_2961 | hypothetical protein | no |
| Mkms_2962 | DNA repair protein RadA | yes |
| Mkms_2963 | hypothetical protein | no |
| Mkms_2964 | hypothetical protein | no |
| Mkms_2965 | putative phage excisionase | yes |
| Mkms_2966 | hypothetical protein | no |
| Mkms_2967 | phage integrase | yes |
