# Supplementary material for: Prophage-like elements present in Mycobacterium genomes
Source: BMC Genomics. 2014 Mar 27;15(1):243. doi: 10.1186/1471-2164-15-243 (PMC3986857; doi:10.1186/1471-2164-15-243)
Supplement: Supplementary file 7 — Additional file 7: Table S7: Database matches for phiMkms_2. (DOC 39 KB) [file 12864_2013_7046_MOESM7_ESM.doc]

Table S7 Database matches for phiMkms_2

| gene | function | Whether it is similar to phage protein |
| --- | --- | --- |
| Mkms_3875 | HNH endonuclease | yes |
| Mkms_3876 | hypothetical protein | no |
| Mkms_3877 | hypothetical protein | yes |
| Mkms_3878 | phage major capsid protein | yes |
| Mkms_3879 | hypothetical protein | no |
| Mkms_3880 | hypothetical protein | no |
| Mkms_3881 | hypothetical protein | no |
| Mkms_3882 | hypothetical protein | no |
| Mkms_3883 | methylmalonyl-CoA mutase | no |
| Mkms_3884 | hypothetical protein | yes |
| Mkms_3885 | excinuclease ABC subunit C | no |
| Mkms_3886 | hypothetical protein | yes |
| Mkms_3887 | hypothetical protein | no |
| Mkms_3888 | HNH endonuclease domain-containing protein | yes |
| Mkms_3889 | hypothetical protein | no |
| Mkms_3890 | PhiRv1 integrase | yes |
