# Supplementary material for: Prophage-like elements present in Mycobacterium genomes
Source: BMC Genomics. 2014 Mar 27;15(1):243. doi: 10.1186/1471-2164-15-243 (PMC3986857; doi:10.1186/1471-2164-15-243)
Supplement: Supplementary file 8 — Additional file 8: Table S8: Database matches for phiBN42_1. (DOC 30 KB) [file 12864_2013_7046_MOESM8_ESM.doc]

Table S8 Database matches for phiBN42_1

| gene | function | Whether it is similar to phage protein |
| --- | --- | --- |
| BN42_21176 | Integrase | yes |
| BN42_21178 | phage transcriptional regulator AlpA/ excisionase | yes |
| BN42_21179 | DNA primase | yes |
| BN42_21180 | hypothetical protein | yes |
| BN42_21182 | phage prohead protease | yes |
| BN42_21183 | phage major capsid protein | yes |
| BN42_21184 | hypothetical protein | yes |
| BN42_21185 | hypothetical protein | no |
