# Supplementary material for: Prophage-like elements present in Mycobacterium genomes
Source: BMC Genomics. 2014 Mar 27;15(1):243. doi: 10.1186/1471-2164-15-243 (PMC3986857; doi:10.1186/1471-2164-15-243)
Supplement: Supplementary file 9 — Additional file 9: Table S9: Database matches for phiBN44_1. (DOC 34 KB) [file 12864_2013_7046_MOESM9_ESM.doc]

Table S9 Database matches for phiBN44_1

| gene | function | Whether it is similar to phage protein |
| --- | --- | --- |
| BN44_60547 | phage major capsid protein | yes |
| BN44_60548 | scaffolding protein | yes |
| BN44_60549 | hypothetical protein | no |
| BN44_60550 | Phage portal protein | yes |
| BN44_60551 | Phage Terminase | yes |
| BN44_60552 | HNH endonuclease | yes |
| BN44_60554 | DNA primase | yes |
| BN44_60555 | Protein of unknown function/DNA polymerase | Human adenovirus |
| BN44_60556 | K+ transporter | no |
| BN44_60557 | XRE family transcriptional regulator | yes |
| BN44_60558 | phage integrase | yes |
