# Supplementary material for: Prophage-like elements present in Mycobacterium genomes
Source: BMC Genomics. 2014 Mar 27;15(1):243. doi: 10.1186/1471-2164-15-243 (PMC3986857; doi:10.1186/1471-2164-15-243)
Supplement: Supplementary file 10 — Additional file 10: Table S10: Database matches for phiMCAN_1. (DOC 33 KB) [file 12864_2013_7046_MOESM10_ESM.doc]

Table S10 Database matches for phiMCAN_1

| gene | function | Whether it is similar to phage protein |
| --- | --- | --- |
| MCAN_10511 | phage integrase | yes |
| MCAN_10521 | DNA-binding protein | yes |
| MCAN_10531 | hypothetical protein | no |
| MCAN_10541 | DNA primase | yes |
| MCAN_10551 | HNH endonuclease | yes |
| MCAN_10561 | phage terminase | yes |
| MCAN_10571 | phage portal protein | yes |
| MCAN_10581 | hypothetical protein | no |
| MCAN_10591 | hypothetical protein | yes |
| MCAN_10601 | phage major capsid protein | yes |
| MCAN_10611 | hypothetical protein | no |
