# Supplementary material for: Prophage-like elements present in Mycobacterium genomes
Source: BMC Genomics. 2014 Mar 27;15(1):243. doi: 10.1186/1471-2164-15-243 (PMC3986857; doi:10.1186/1471-2164-15-243)
Supplement: Supplementary file 11 — Additional file 11: Table S11: Ddatabase matches for phiMycsm_1. (DOC 34 KB) [file 12864_2013_7046_MOESM11_ESM.doc]

Table S11 Database matches for phiMycsm_1

| gene | function | Whether it is similar to phage protein |
| --- | --- | --- |
| Mycsm_04291 | [single-stranded DNA-binding protein](http://blast.ncbi.nlm.nih.gov/Blast.cgi#alnHdr_403251176) | yes |
| Mycsm_04292 | hypothetical protein | no |
| Mycsm_04293 | glycerate kinase | no |
| Mycsm_04294 | hypothetical protein | yes |
| Mycsm_04295 | hypothetical protein | yes |
| Mycsm_04296 | phage major capsid protein | yes |
| Mycsm_04297 | hypothetical protein | yes |
| Mycsm_04298 | Phage portal protein | yes |
| Mycsm_04299 | phage terminase | yes |
| Mycsm_04300 | hypothetical protein | no |
| Mycsm_04301 | hypothetical protein | no |
| Mycsm_04302 | phage transcriptional regulator AlpA | yes |
| Mycsm_04303 | integrase | yes |
