# Supplementary material for: Prophage-like elements present in Mycobacterium genomes
Source: BMC Genomics. 2014 Mar 27;15(1):243. doi: 10.1186/1471-2164-15-243 (PMC3986857; doi:10.1186/1471-2164-15-243)
Supplement: Supplementary file 12 — Additional file 12: Table S12: Database matches for phiW7S_1. (DOC 34 KB) [file 12864_2013_7046_MOESM12_ESM.doc]

Table S12 Database matches for phiW7S_1

| gene | function | Whether it is similar to phage protein |
| --- | --- | --- |
| W7S_04825 | phage integrase | yes |
| W7S_04830 | hypothetical protein | no |
| W7S_04835 | hypothetical protein | yes |
| W7S_04840 | hypothetical protein | yes |
| W7S_04845 | pantothenate kinase | yes |
| W7S_04850 | Mg2+ transporter protein, CorA-like protein | no |
| W7S_04855 | transposase | yes |
| W7S_04860 | transposase | no |
| W7S_04865 | IstB domain-containing protein ATP-binding protein | no |
| W7S_04870 | hypothetical protein | no |
| W7S_04875 | hypothetical protein | yes |
| W7S_04880 | hypothetical protein | no |
