# Supplementary material for: Prophage-like elements present in Mycobacterium genomes
Source: BMC Genomics. 2014 Mar 27;15(1):243. doi: 10.1186/1471-2164-15-243 (PMC3986857; doi:10.1186/1471-2164-15-243)
Supplement: Supplementary file 13 — Additional file 13: Figure S1-S11: Comparative genomic analyses of phi172_2 and cluster A (subcluster A1-A11) mycobacteriophage. (DOC 5 MB) [file 12864_2013_7046_MOESM13_ESM.doc]

**Additional file 13 –Figure S1-S11.** Comparative genomic analyses of phi172_2 and cluster A (subclusterA1-A11) mycobacteriophage

phiM172_2

phiM172_2


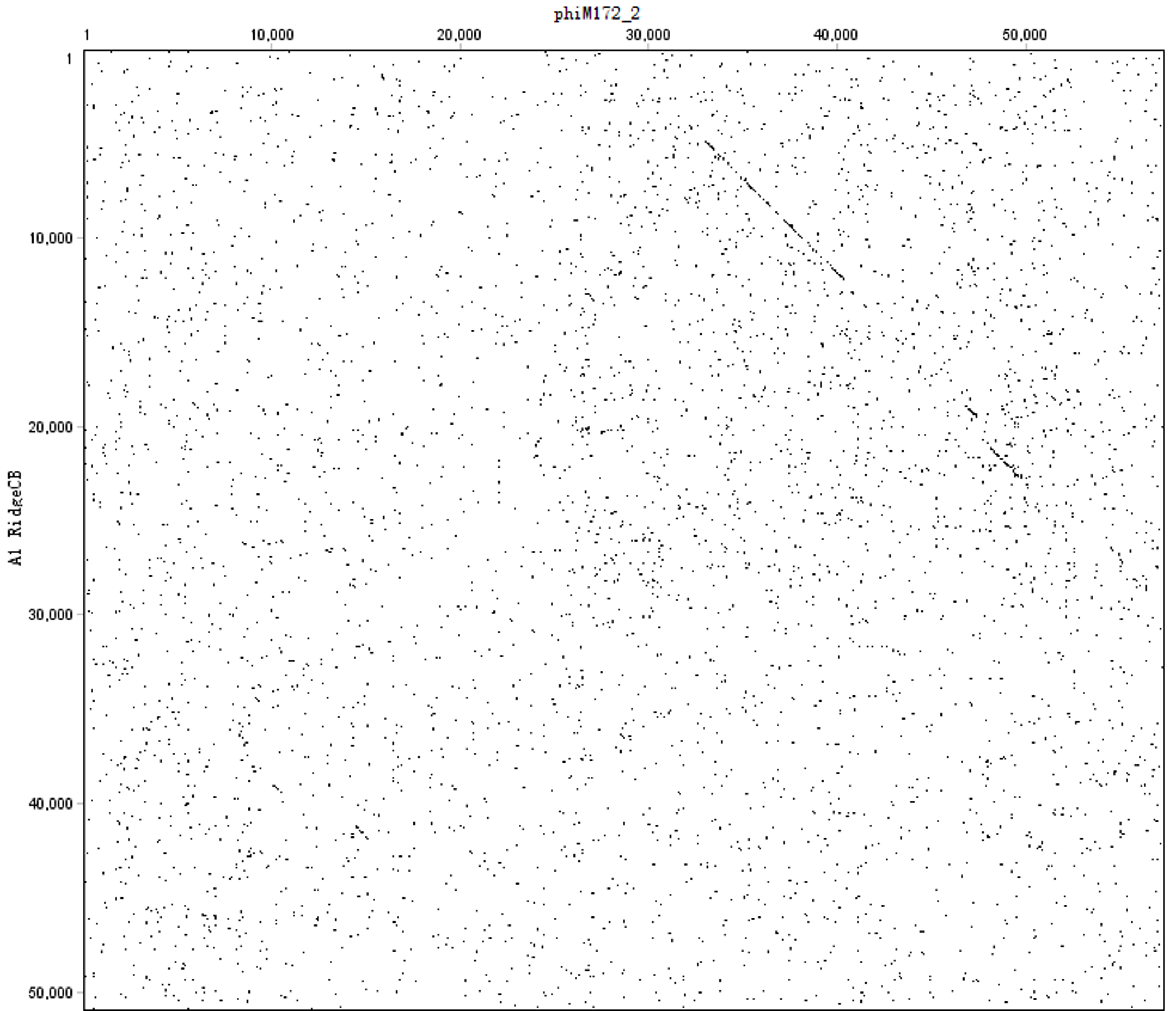

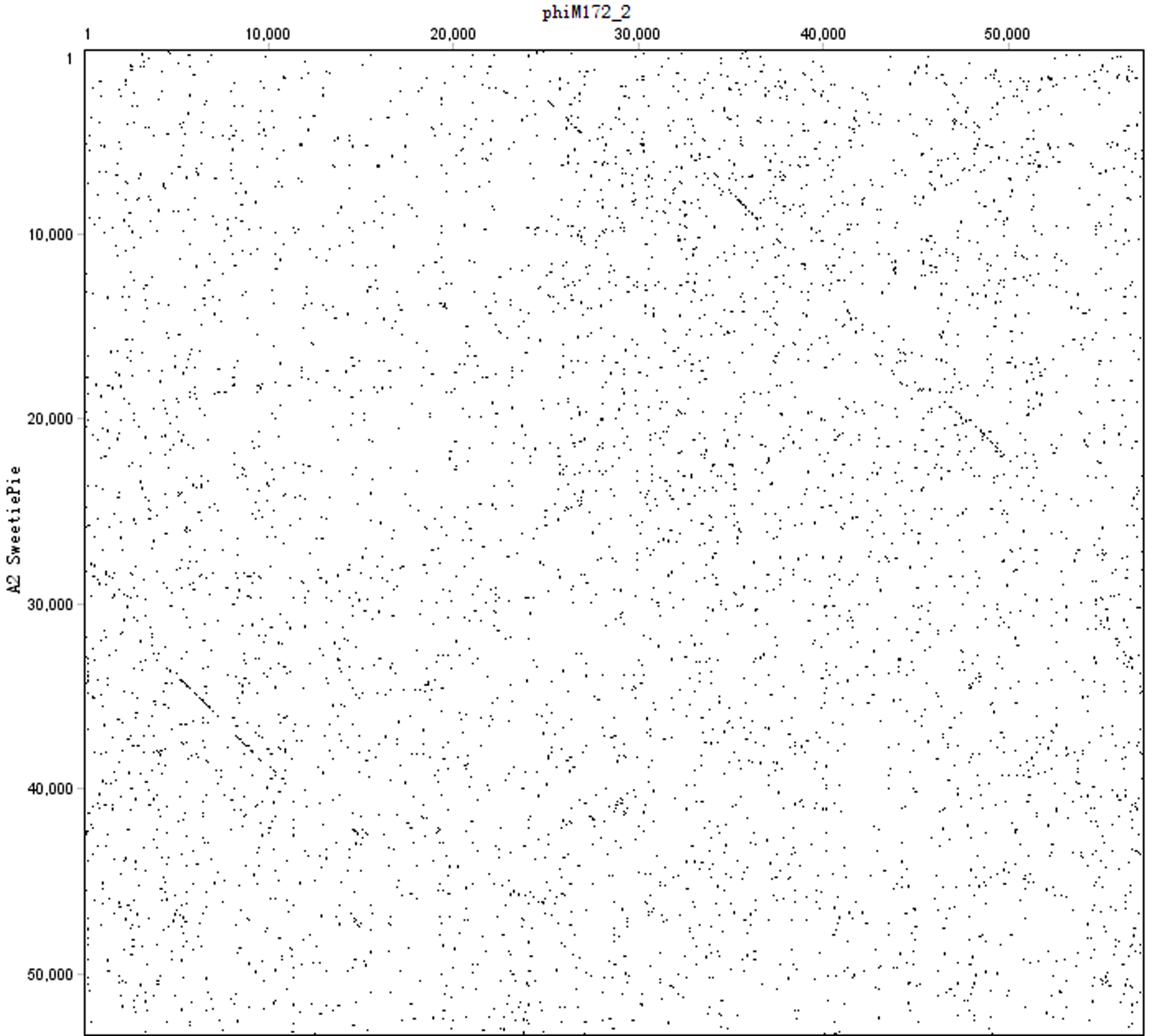


subclusterA1

subclusterA2

phiM172_2

phiM172_2


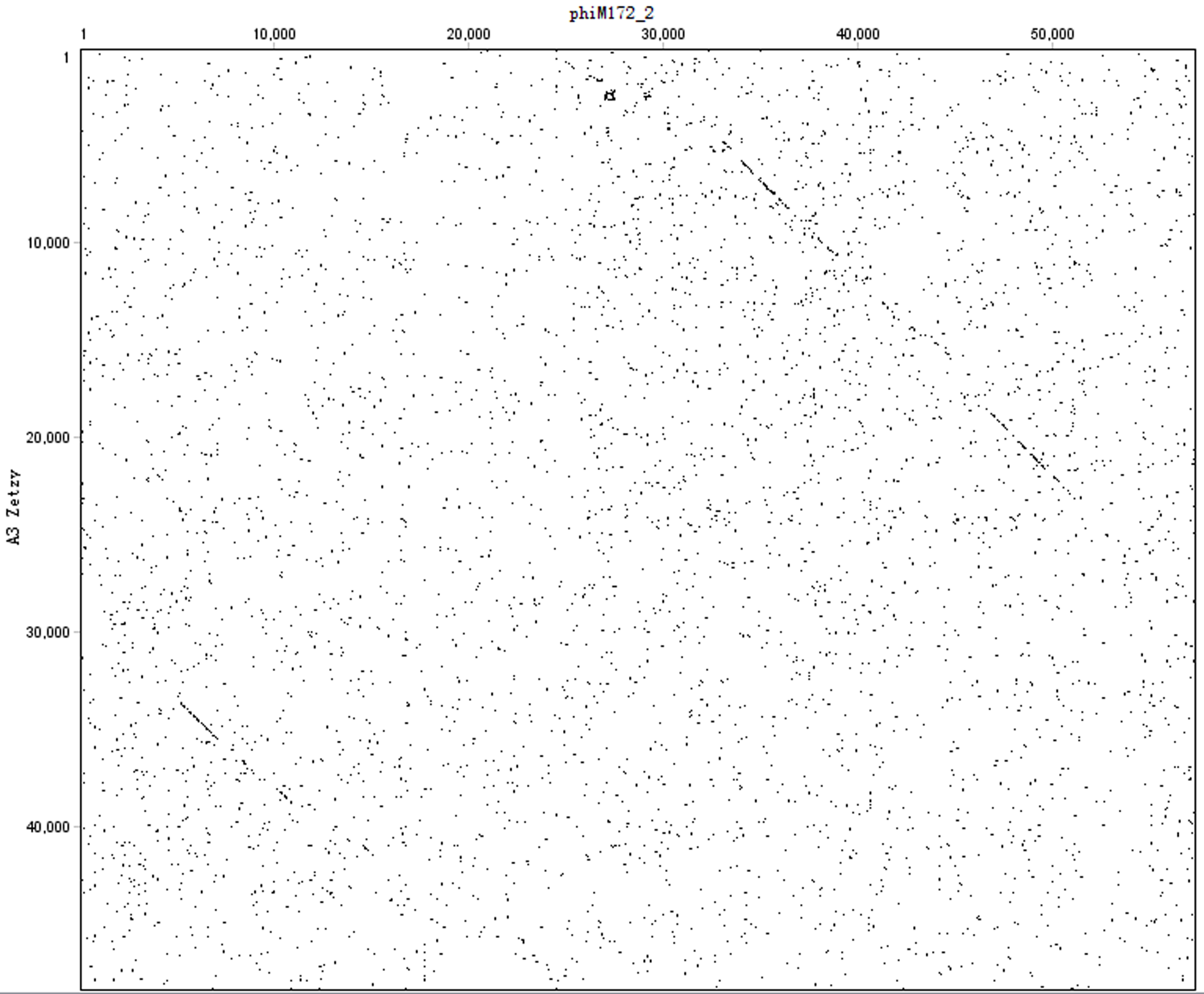

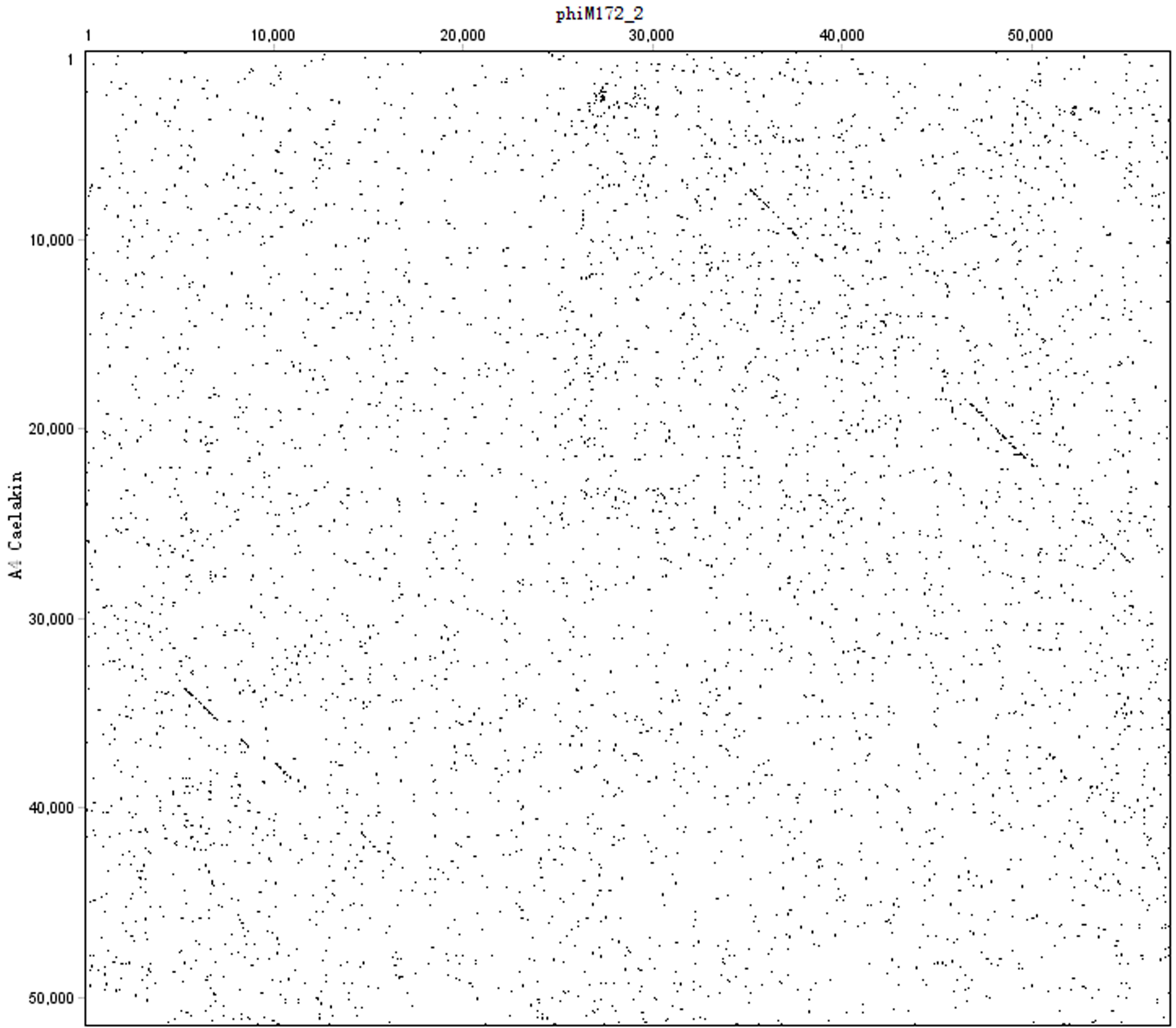


subclusterA3

subclusterA4


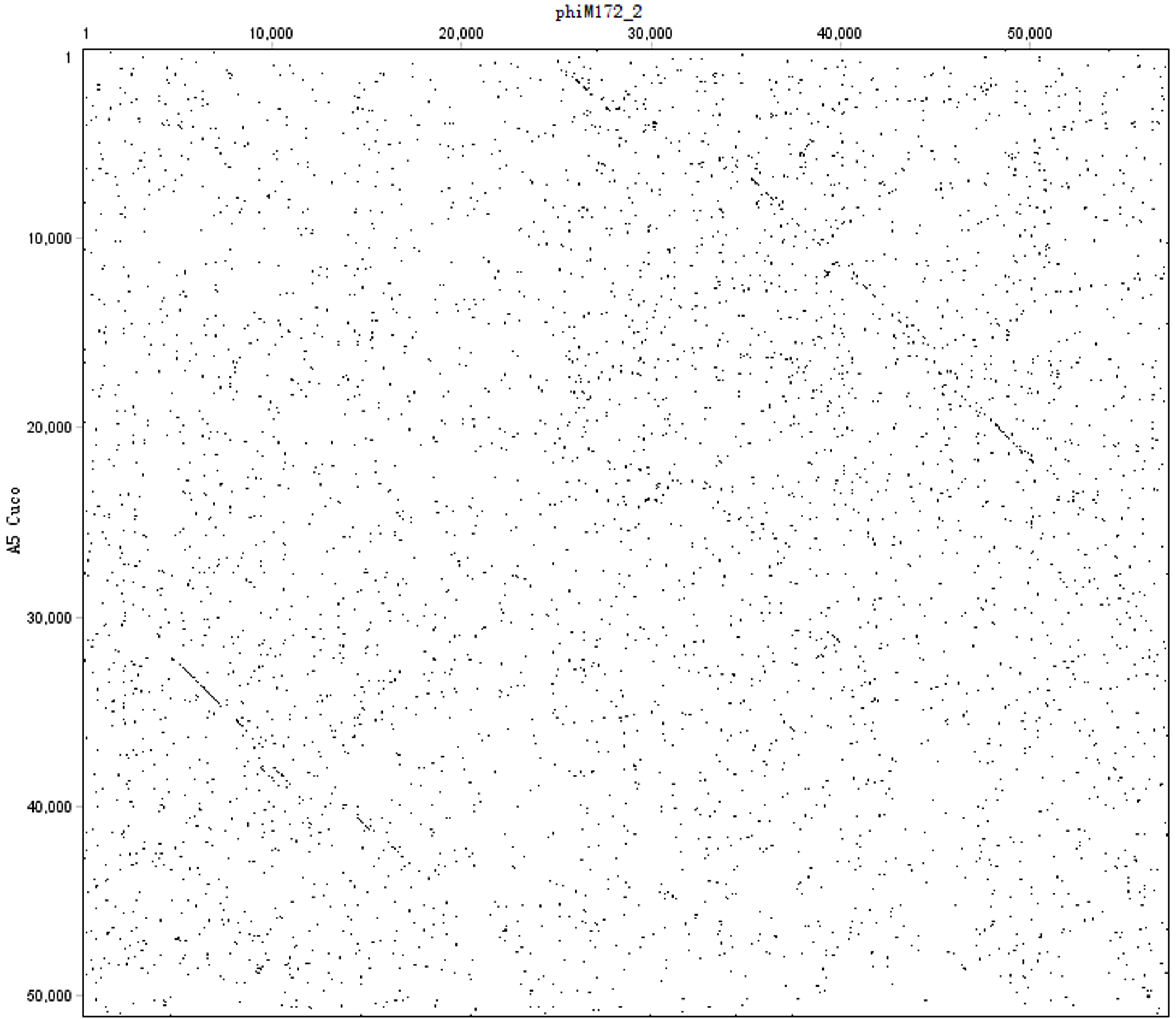

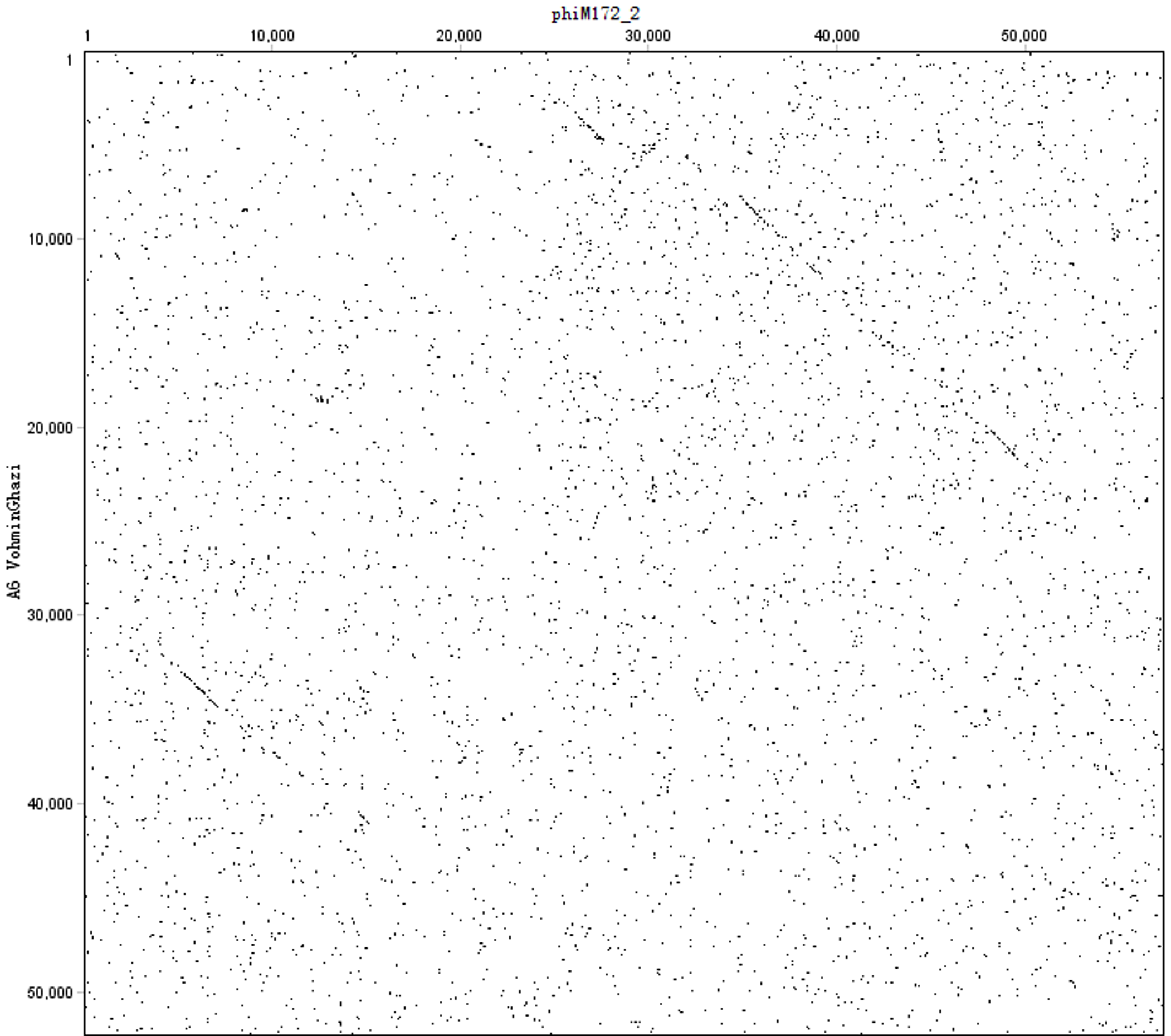


phiM172_2

phiM172_2

subclusterA5

subclusterA6

phiM172_2

phiM172_2


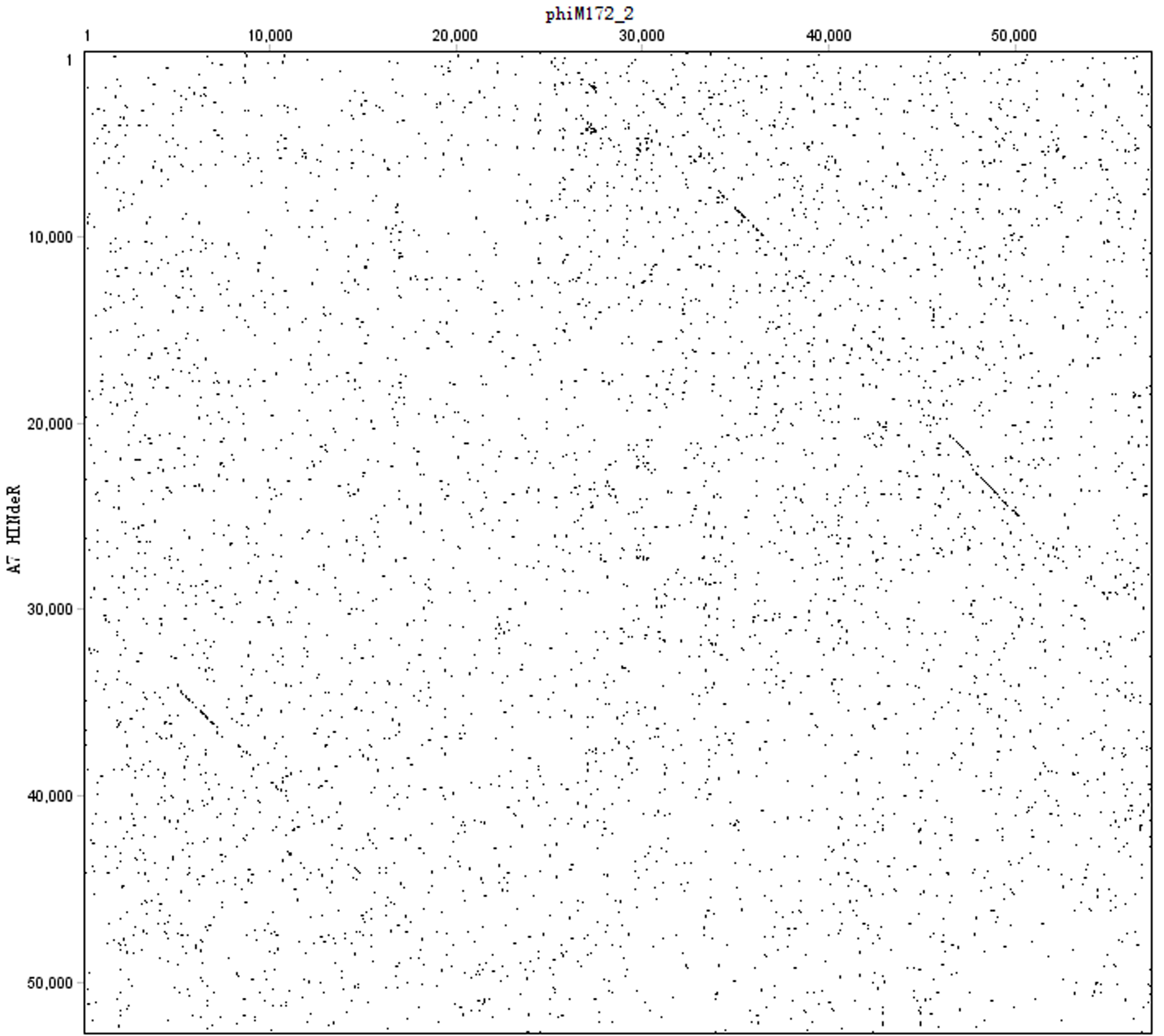

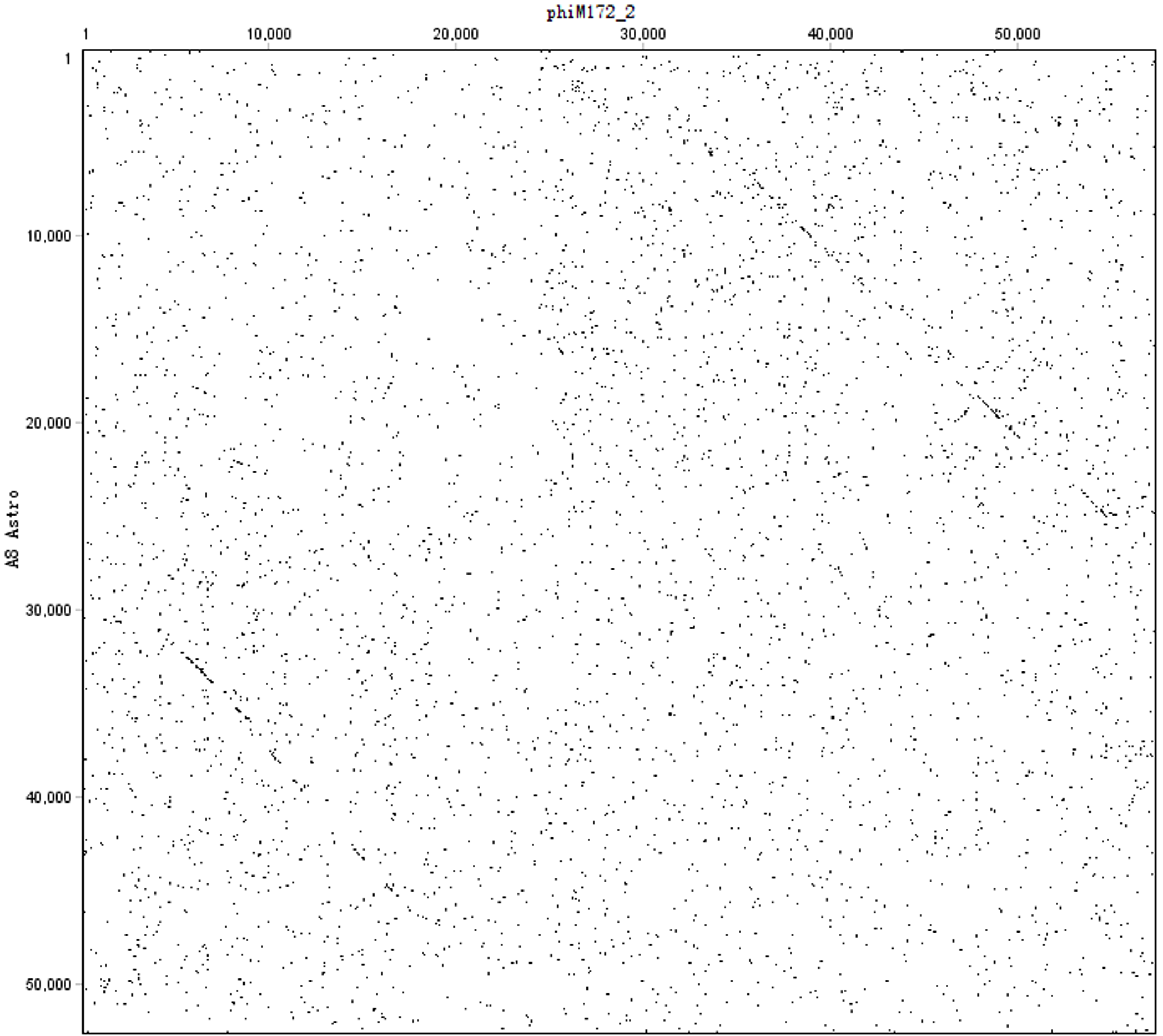


subclusterA7

subclusterA8

phiM172_2

phiM172_2


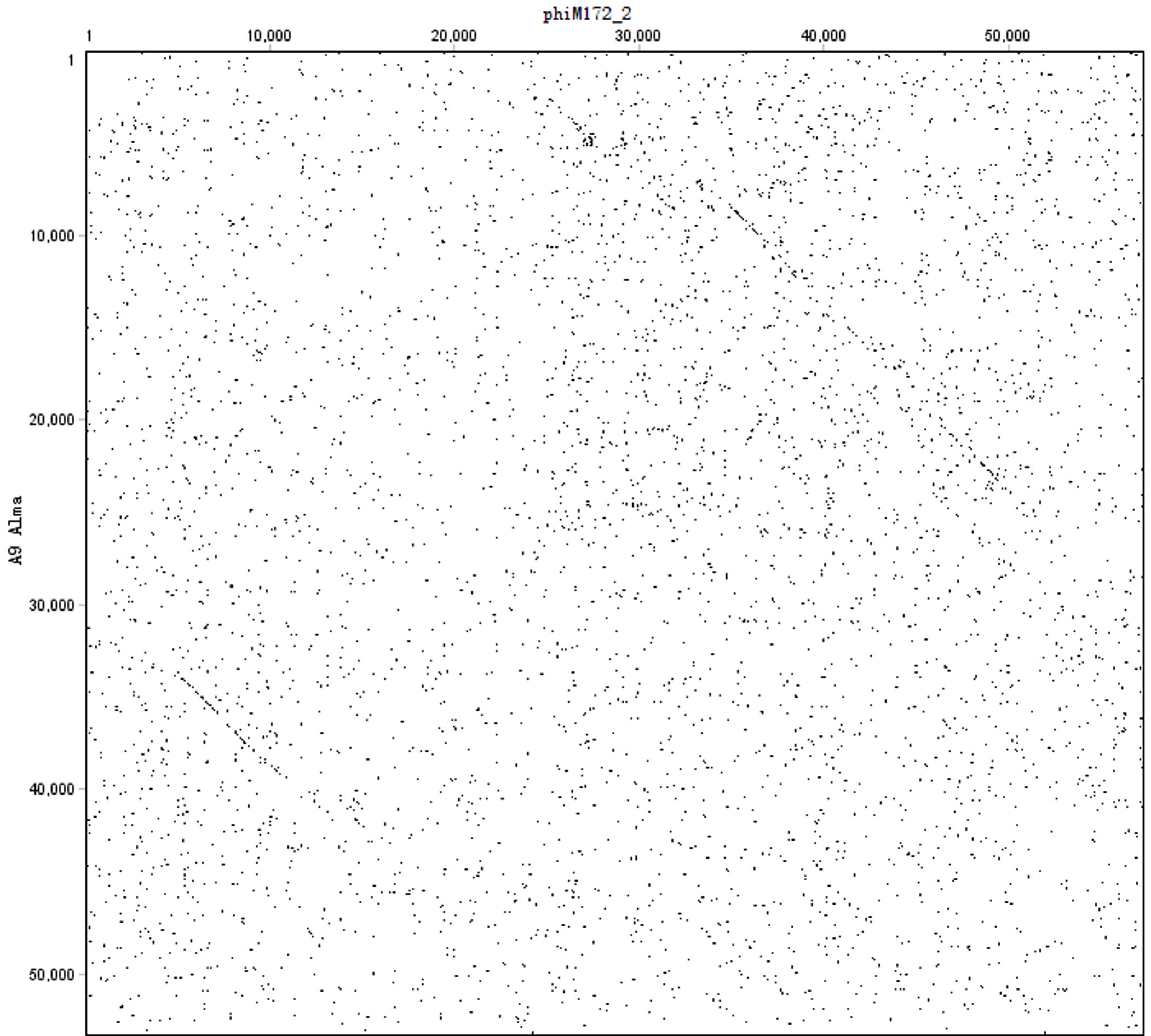

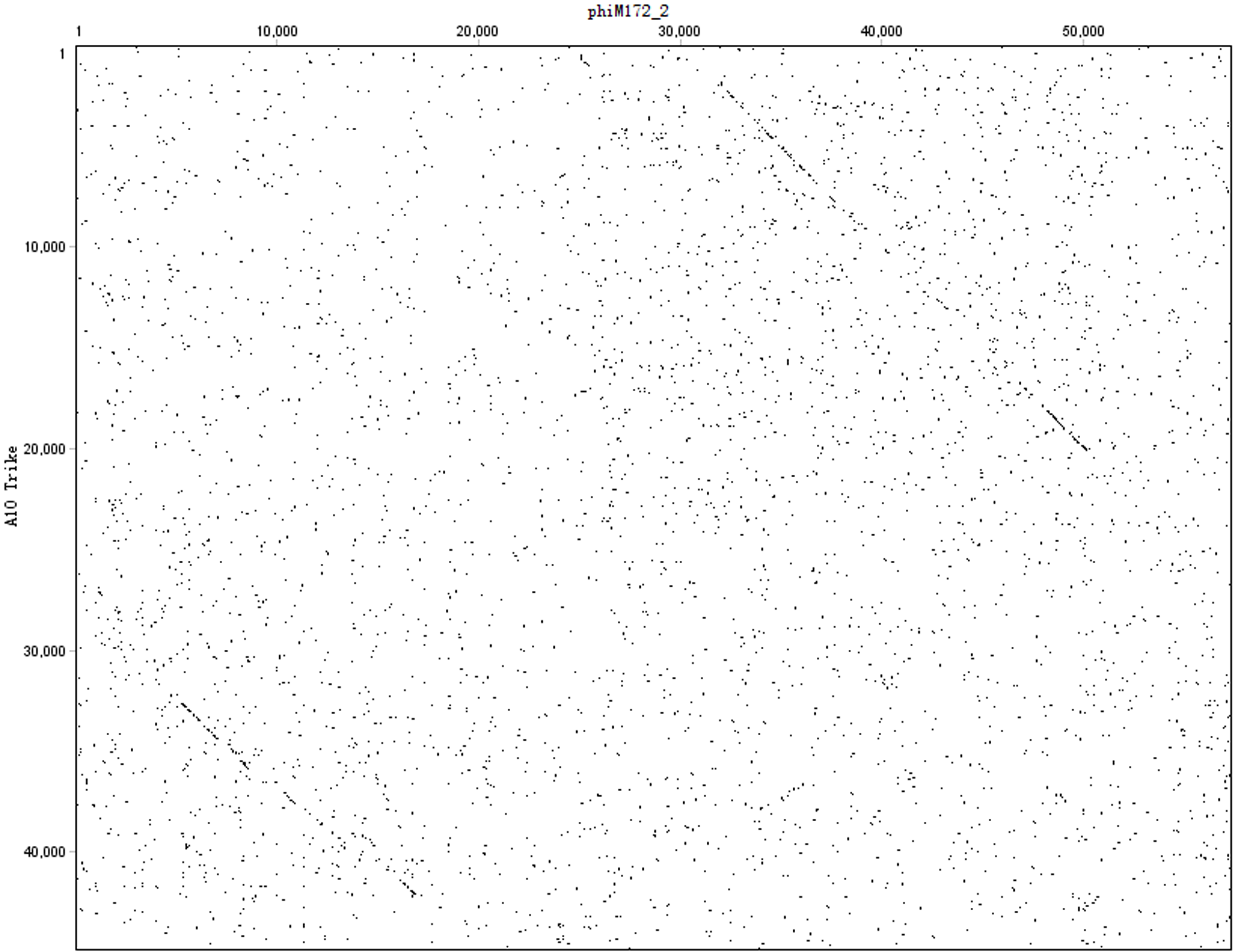


subclusterA9

subclusterA10

phiM172_2


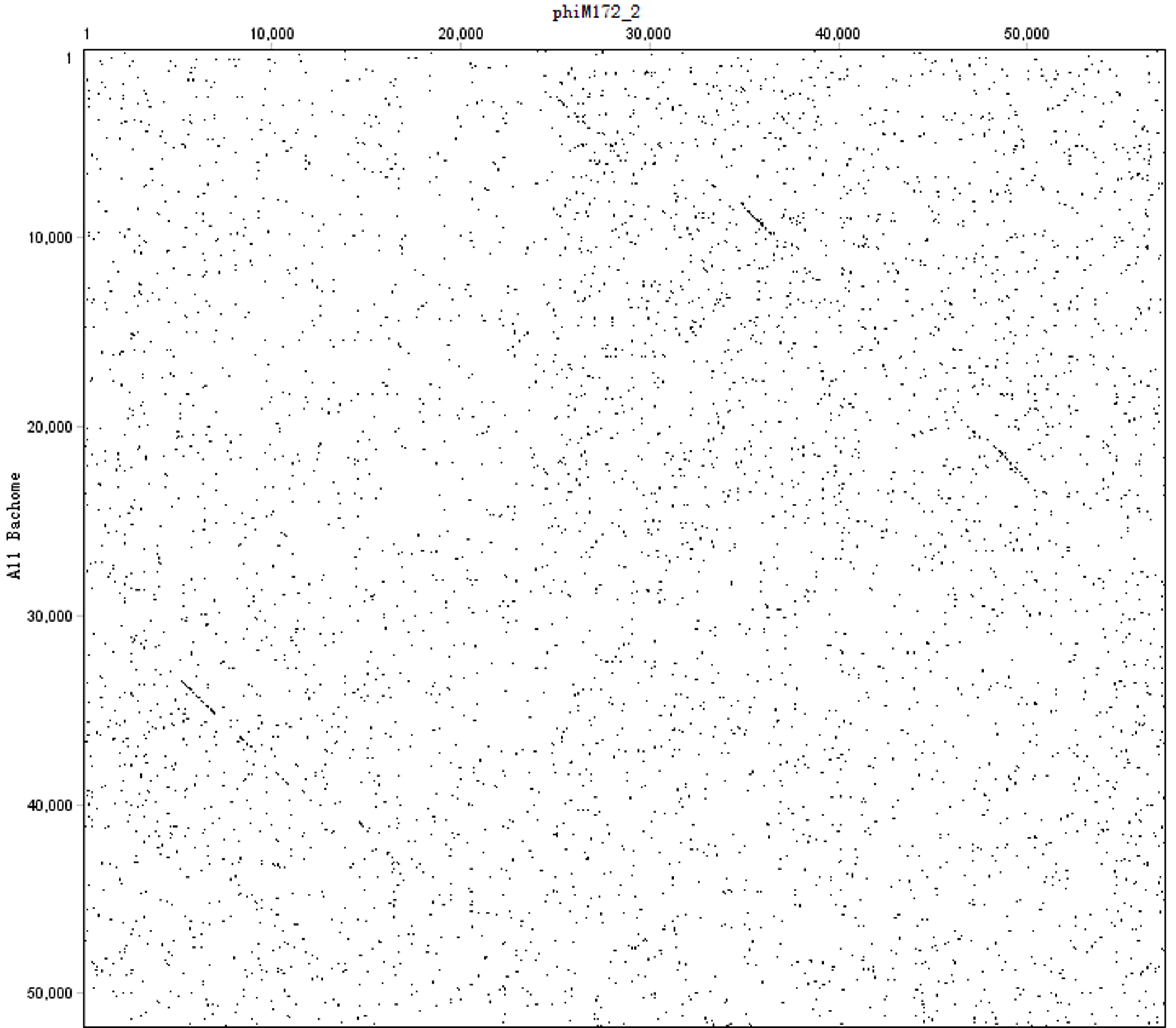


subclusterA11
