# Supplementary material for: Prophage-like elements present in Mycobacterium genomes
Source: BMC Genomics. 2014 Mar 27;15(1):243. doi: 10.1186/1471-2164-15-243 (PMC3986857; doi:10.1186/1471-2164-15-243)
Supplement: Supplementary file 14 — Additional file 14: Figure S12: Comparative genomic analyses of phiMAB_1 and subcluster F1 mycobacteriophage. (DOC 549 KB) [file 12864_2013_7046_MOESM14_ESM.doc]

**Additional file 14 –Figure S12.** Comparative genomic analyses of phiMAB_1 and subcluster F1 mycobacteriophage

**
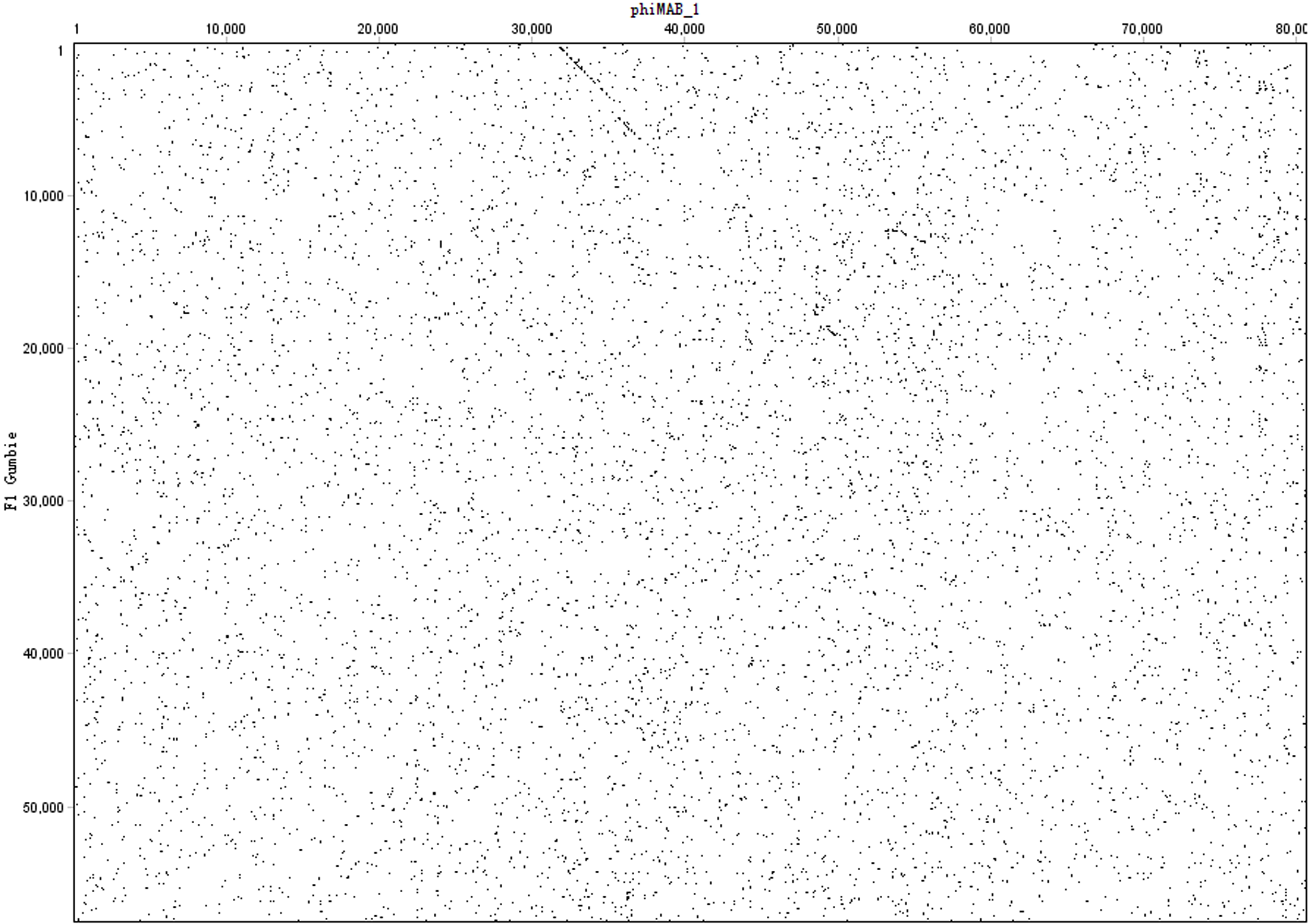
**

phiMAB_1

subcluster F1
