# Supplementary material for: Prophage-like elements present in Mycobacterium genomes
Source: BMC Genomics. 2014 Mar 27;15(1):243. doi: 10.1186/1471-2164-15-243 (PMC3986857; doi:10.1186/1471-2164-15-243)
Supplement: Supplementary file 15 — Additional file 15: Figure S13-S14: Comparative genomic analyses of phiMAB47J26_1, subcluster F1 and cluster N mycobacteriophage. (DOC 1 MB) [file 12864_2013_7046_MOESM15_ESM.doc]

**Additional file 15 –Figure S13-S14.** Comparative genomic analyses of phiMAB47J26_1, subcluster F1 and cluster N mycobacteriophage


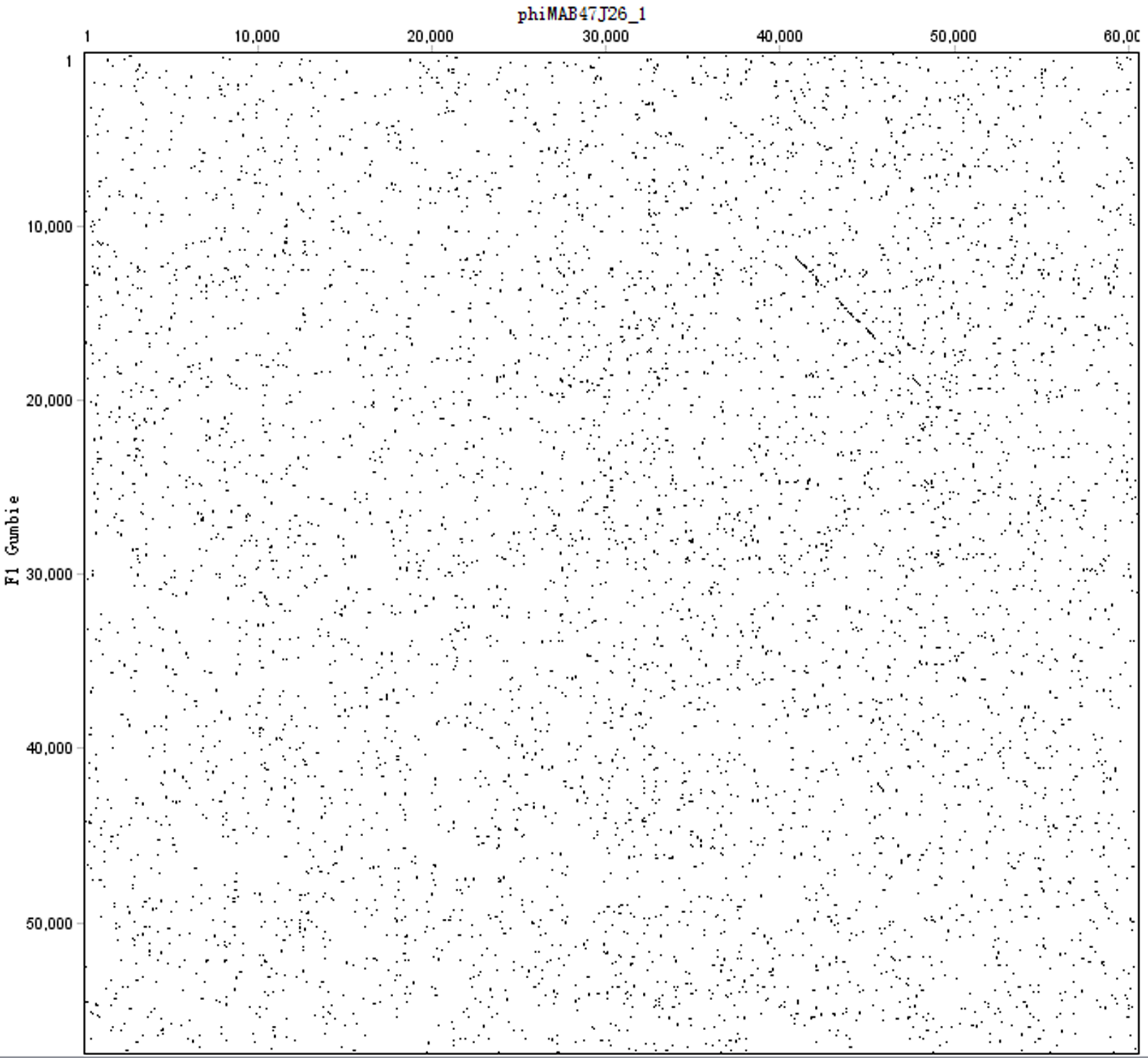

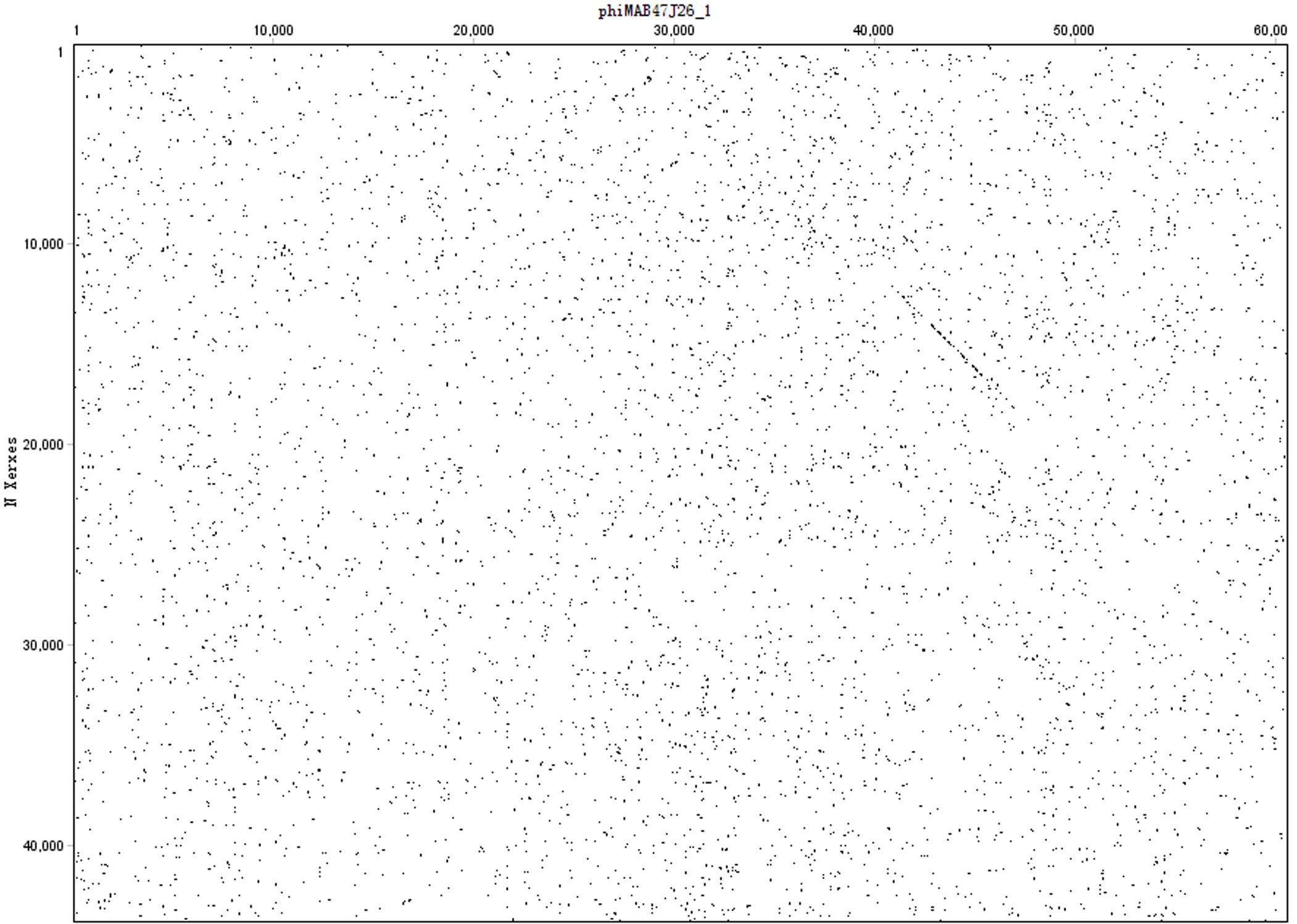


phiMAB47J26_1**1**

subcluster F1

phiMAB47J26_1**1**

cluster N
