# Supplementary material for: Prophage-like elements present in Mycobacterium genomes
Source: BMC Genomics. 2014 Mar 27;15(1):243. doi: 10.1186/1471-2164-15-243 (PMC3986857; doi:10.1186/1471-2164-15-243)
Supplement: Supplementary file 16 — Additional file 16: Figure S15-S17: Comparative genomic analyses of phiMAB47J26_2, cluster P, subcluster F1 and cluster N mycobacteriophage. (DOC 2 MB) [file 12864_2013_7046_MOESM16_ESM.doc]

**Additional file 16 –Figure S15-S17.** Comparative genomic analyses of phiMAB47J26_2, cluster P, subcluster F1 and cluster N mycobacteriophage


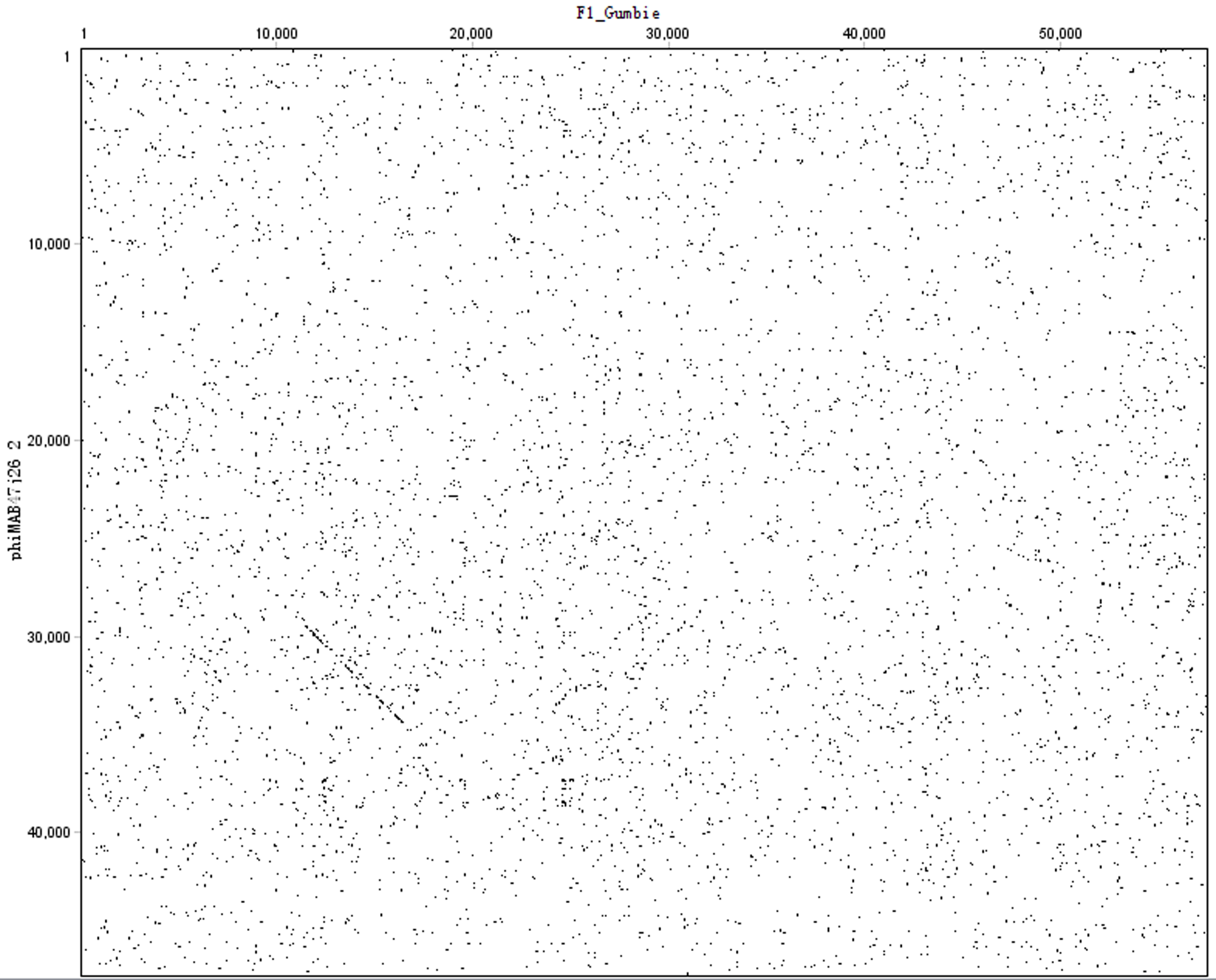

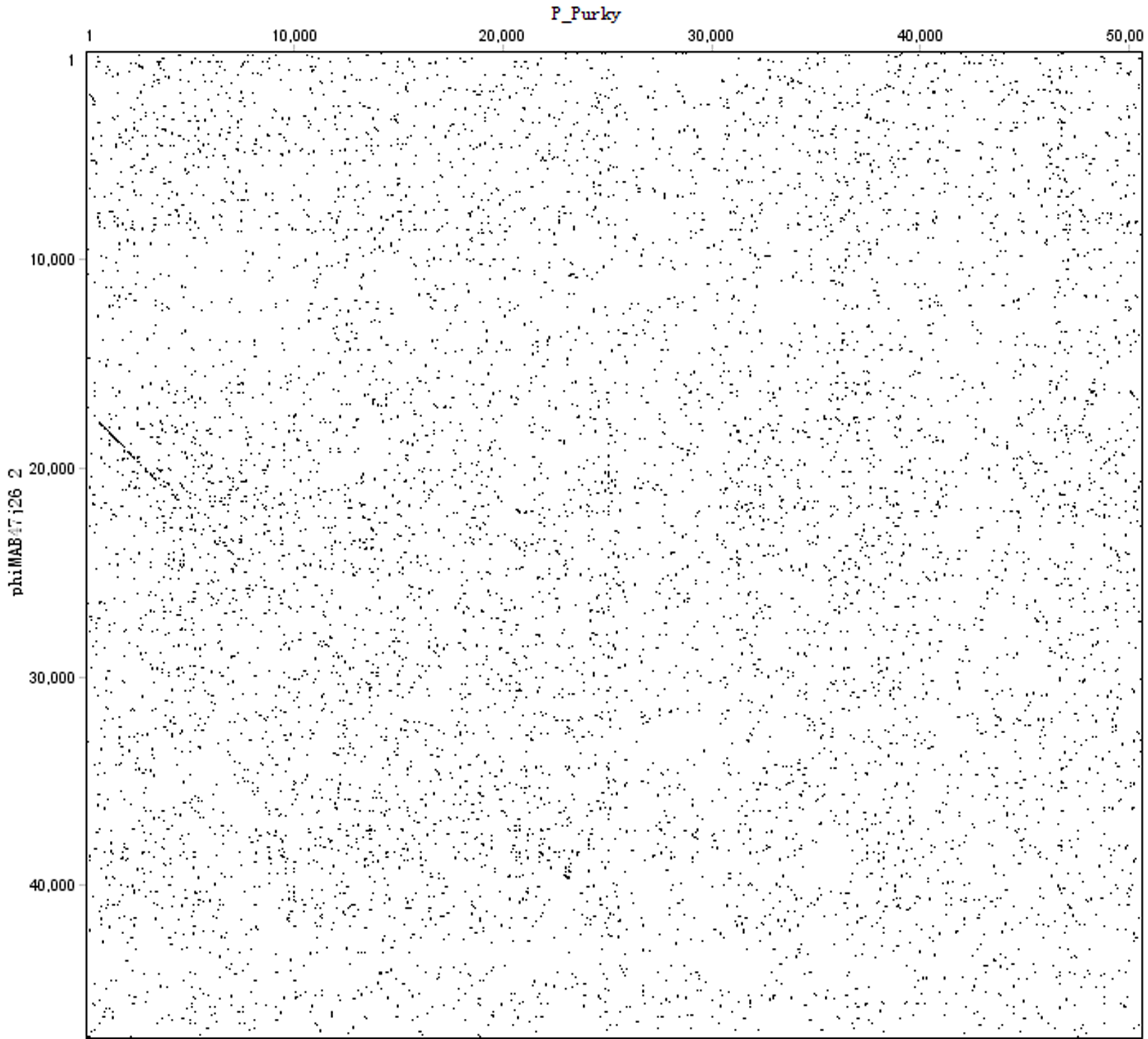

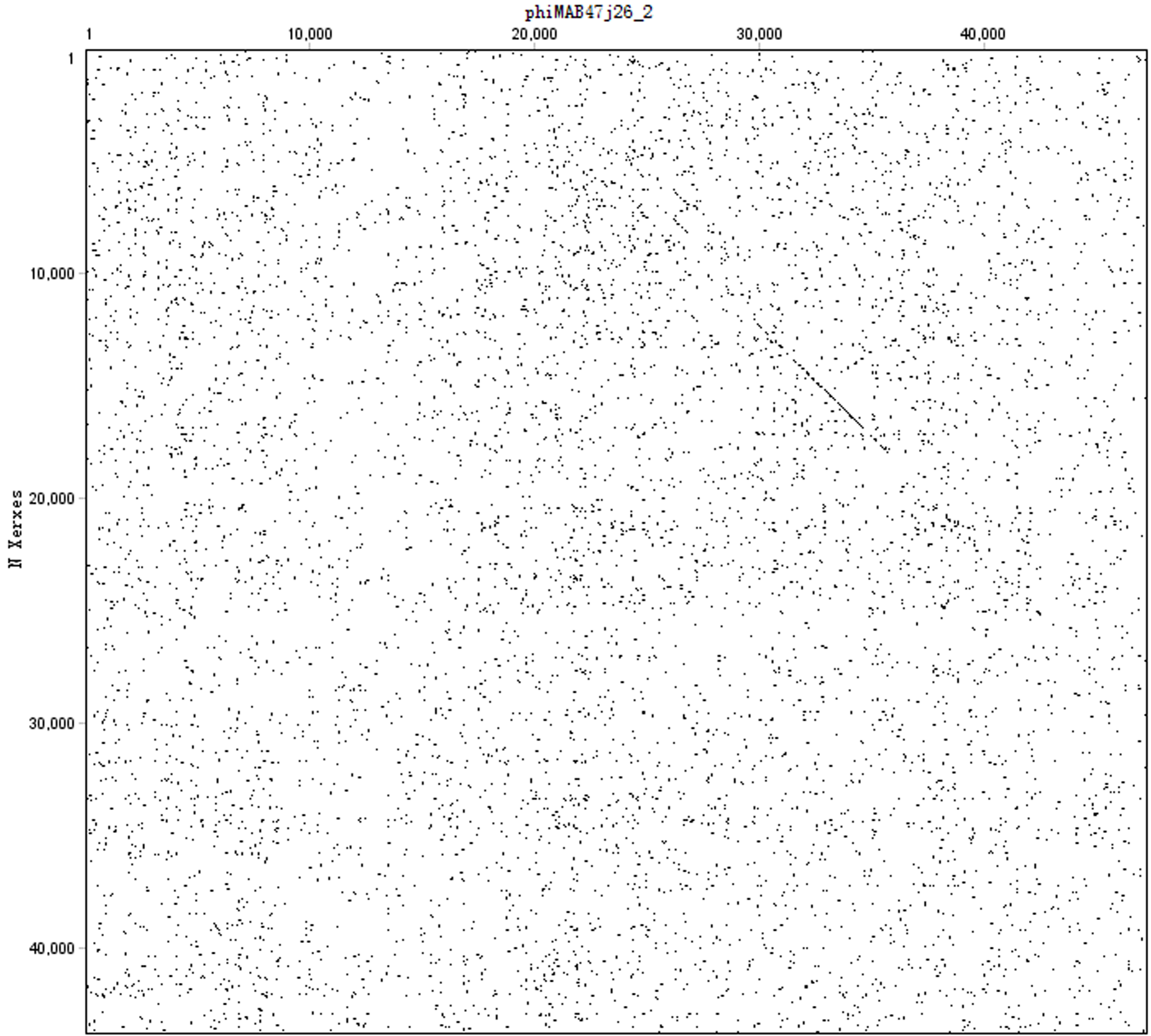


phiMAB47J26_2**1**

subcluster F1

phiMAB47J26_2**1**

cluster P

phiMAB47J26_2**1**

cluster N
