# Supplementary material for: Prophage-like elements present in Mycobacterium genomes
Source: BMC Genomics. 2014 Mar 27;15(1):243. doi: 10.1186/1471-2164-15-243 (PMC3986857; doi:10.1186/1471-2164-15-243)
Supplement: Supplementary file 17 — Additional file 17: Figure S18-S19: Comparative genomic analyses of phi172_1, subcluster F1 and cluster N mycobacteriophage. (DOC 1 MB) [file 12864_2013_7046_MOESM17_ESM.doc]

**Additional file 17 –Figure S18-S19.** Comparative genomic analyses of phi172_1,subcluster F1 and cluster N mycobacteriophage


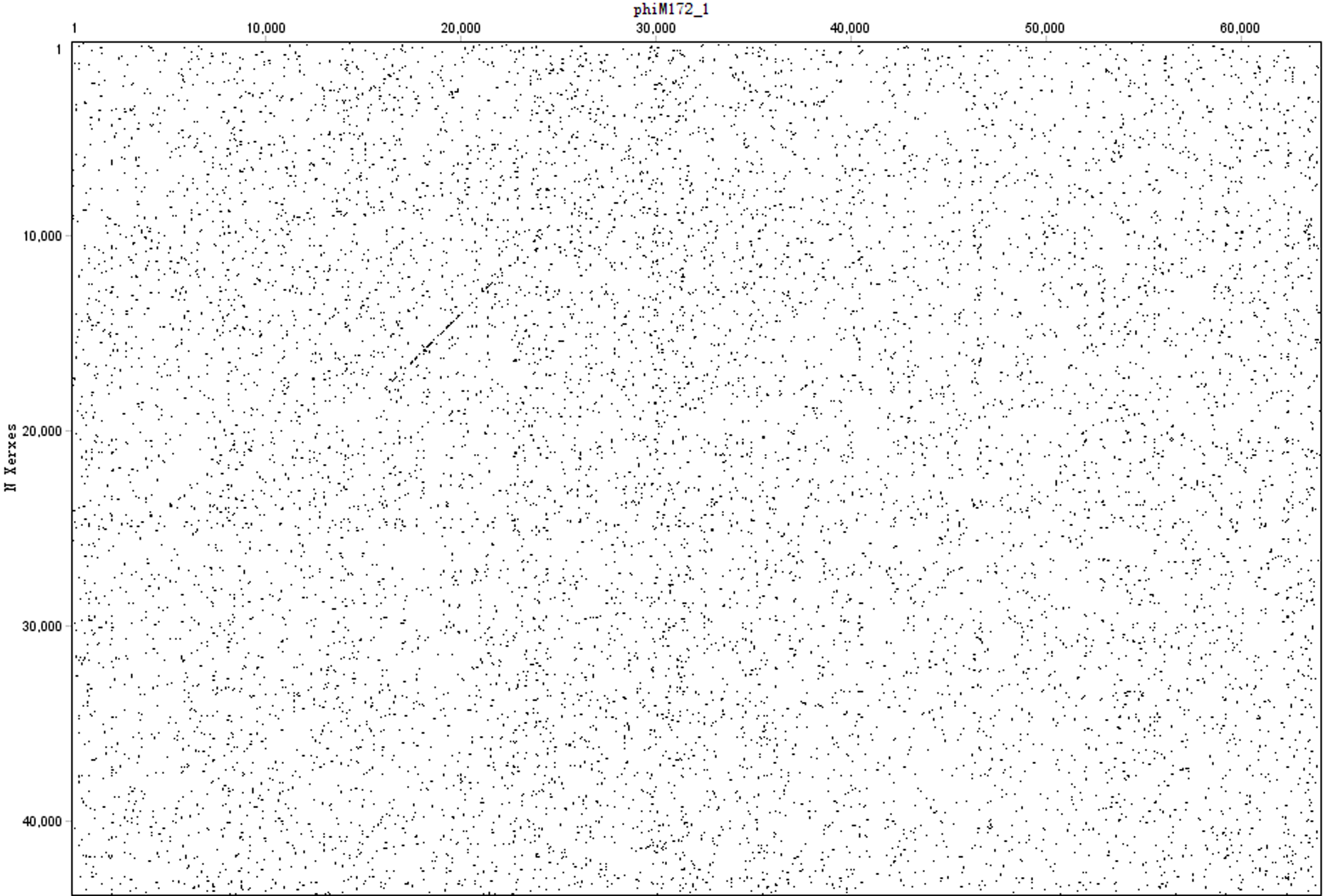

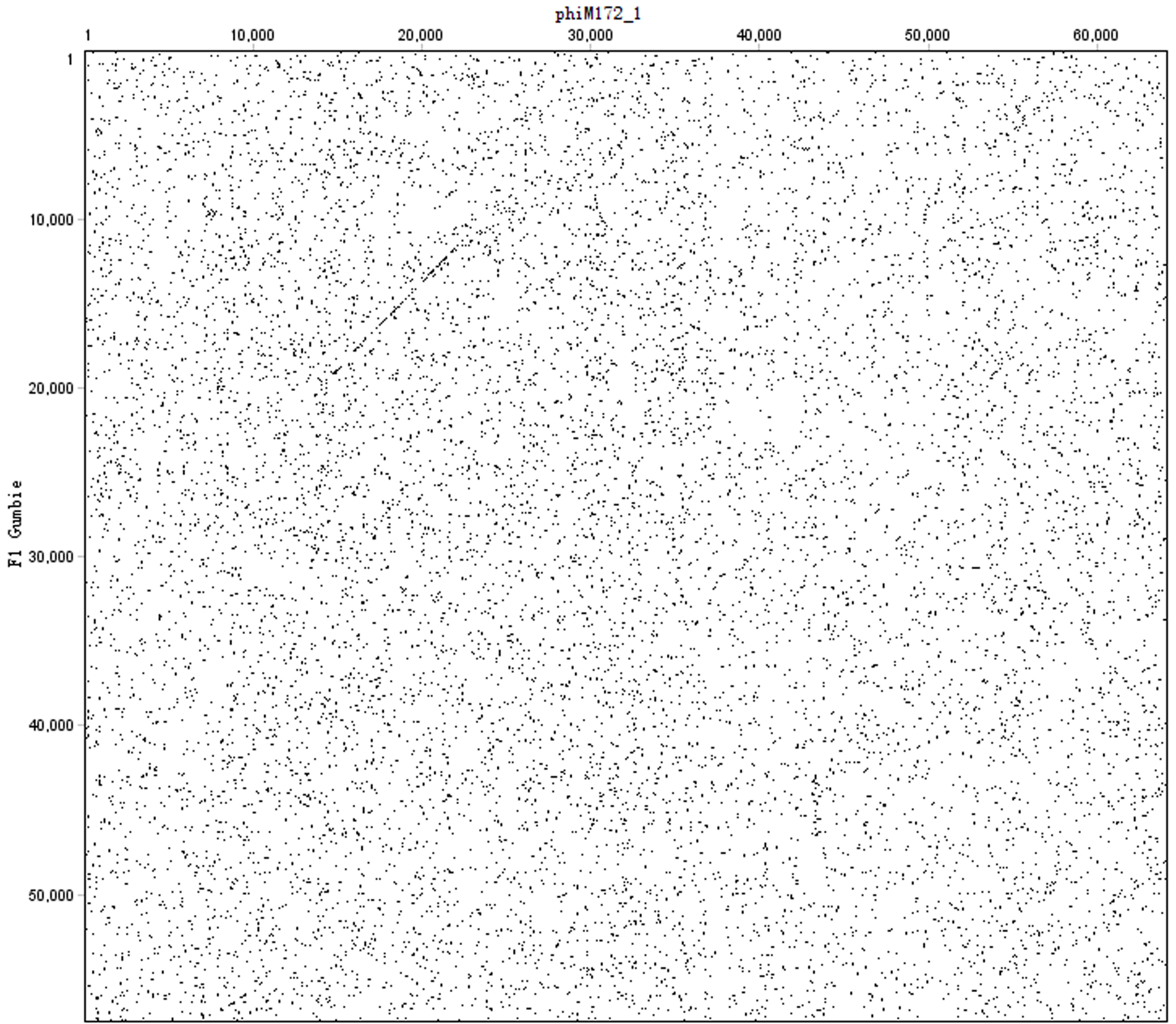


phi172_1

subcluster F1

phi172_1

**1**

cluster N
